# High-dimensional analysis of NK cells in kidney transplantation uncovers subsets associated with antibody-independent graft dysfunction

**DOI:** 10.1172/jci.insight.185687

**Published:** 2024-11-08

**Authors:** Dan Fu Ruan, Miguel Fribourg, Yuko Yuki, Yeon-Hwa Park, Maureen P. Martin, Haocheng Yu, Geoffrey C. Kelly, Brian Lee, Ronaldo M. de Real, Rachel Lee, Daniel Geanon, Seunghee Kim-Schulze, Nicholas Chun, Paolo Cravedi, Mary Carrington, Peter S. Heeger, Amir Horowitz

**Affiliations:** 1 Department of Immunology and Immunotherapy,; 2 Department of Oncological Sciences,; 3 The Marc and Jennifer Lipschultz Precision Immunology Institute,; 4 Tisch Cancer Institute, and; 5 Division of Nephrology, Department of Medicine, Translational Transplant Research Center, Icahn School of Medicine at Mount Sinai, New York, New York, USA.; 6 Ragon Institute of MGH, MIT and Harvard, Cambridge, Massachusetts, USA.; 7 Basic Science Program, Frederick National Laboratory for Cancer Research, National Cancer Institute, Frederick, Maryland, USA.; 8 Laboratory of Integrative Cancer Immunology, Center for Cancer Research, National Cancer Institute, Bethesda, Maryland, USA.; 9 Human Immune Monitoring Center, Icahn School of Medicine at Mount Sinai, New York, New York, USA.; 10 Department of Medicine, Cedars-Sinai Medical Center, Los Angeles, California, USA.

**Keywords:** Immunology, Transplantation, NK cells, Organ transplantation

## Abstract

Natural killer (NK) cells respond to diseased and allogeneic cells through NKG2A/HLA-E or killer cell immunoglobulin-like receptor (KIR)/HLA-ABC interactions. Correlations between HLA/KIR disparities and kidney transplant pathology suggest an antibody-independent pathogenic role for NK cells in transplantation, but the mechanisms remain unclear. Using CyTOF to characterize recipient peripheral NK cell phenotypes and function, we observed diverse NK cell subsets among participants who responded heterogeneously to allo-stimulators. NKG2A^+^KIR^+^ NK cells responded more vigorously than other subsets, and this heightened response persisted after kidney transplantation despite immunosuppression. In test and validation sets from 2 clinical trials, pretransplant donor-induced release of cytotoxicity mediator Ksp37 by NKG2A^+^ NK cells correlated with reduced long-term allograft function. Separate analyses showed that *Ksp37* gene expression in allograft biopsies lacking histological rejection correlated with death-censored graft loss. Our findings support an antibody-independent role for NK cells in transplant injury and support further testing of pretransplant, donor-reactive, NK cell–produced Ksp37 as a risk-assessing, transplantation biomarker.

## Abstract 2

We performed CyTOF analyses of blood-derived NK cells from two kidney transplant cohorts to profile the heterogeneity and pathogenic function of alloreactive recipient NK cells.

## Graphical abstract

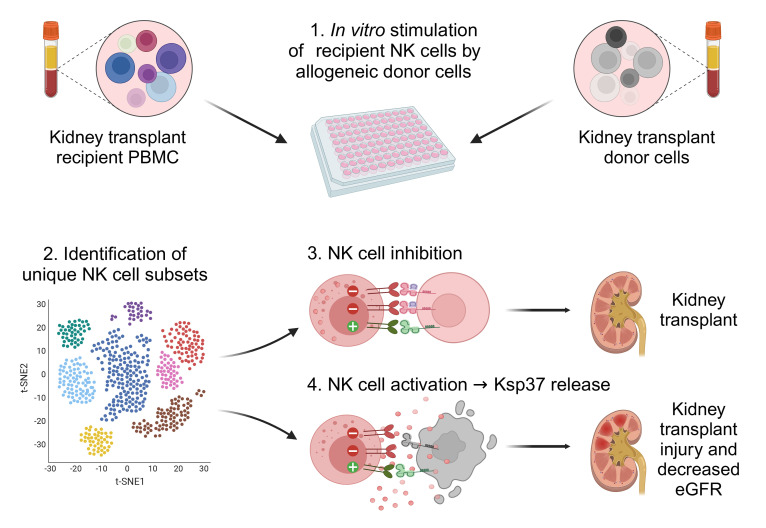

## Introduction

Human natural killer (NK) cells are a population of cytotoxic lymphocytes that participate in cancer surveillance, viral infection control, and contribute to the pathogenesis of graft-versus-host disease and solid organ transplant rejection. NK cell development and function depend on an array of polymorphic receptors that transmit activating or inhibitory signals. Following release from the bone marrow, immature NK cells undergo a developmental self-education program instructed by cognate interactions between various inhibitory NK cell receptors and their ligands. This process instructs NK cells to distinguish diseased from healthy autologous cells and augments their functional capacity. This educational process involves 3 genomic complexes: class I HLA, which encode the A3, A11, Bw4, C1, and C2 ligands; the NK complex (lectin-like CD94 and NKG2A); and the leukocyte receptor complex (killer cell immunoglobulin-like receptors [KIRs]) (1–3). NKG2A interacts with HLA-E, whereas the various KIRs ligate epitopes presented on all HLA-C alleles and most HLA-A and HLA-B alleles (4–7). Activating receptor-ligand interactions that trigger effector functions, but are not involved in education, include NKG2D, DNAM-1, and the natural cytotoxicity receptors.

Traditionally, NK cells have been divided into subsets based on surface expression levels of the CD56 adhesion molecule and the CD16 Fc receptor (FcγRIII). CD56 is acquired in early NK cell development and is highly expressed in the immature CD56^bright^ NK cells compared with the more mature CD56^dim^ subset. CD56^bright^ NK cells are less cytotoxic than CD56^dim^ NK cells, but are strong producers of cytokines, including IFN-γ and TNF-α, induced in response to stimulation with IL-2, IL-12, and IL-18. As CD56^bright^ NK cells mature into CD56^dim^ NK cells, they upregulate surface expression of educating KIRs and acquire larger stores of cytotoxic molecules, including perforin, granzyme B, and Ksp37 (8, 9). Terminally mature CD56^dim^ NK cells acquire CD57 expression (10). The majority of CD16^+^ NK cells are found in the CD56^dim^ NK cell subset, where CD16 facilitates antibody-dependent cellular cytotoxicity (ADCC) (11, 12). While categorizing NK cells based on CD56/CD16 has historic utility, studies published since 2013 uncovered the enormous phenotypic heterogeneity of NK cells and the complex regulation of NK cell function by HLA/KIR education, which together impact functional capacity in various contexts. Mass cytometry analyses revealed up to 30,000 phenotypic populations of NK cells wherein both genetics and environment, e.g., prior exposure to pathogens, shape the diversity of the NK cell compartment (13).

In kidney transplantation, evidence indicates that CD56^dim^CD16^+^ NK cells with the capacity to perform ADCC are crucial pathogenic mediators underlying antibody-mediated rejection (ABMR). Enrichment of NK cells producing cytotoxic molecules, including granulysin, perforin, and Ksp37, are detectable in ABMR lesions with and without serum donor-specific antibodies (DSAs) (14–16). Multiplex immunofluorescent staining and single-cell RNA sequencing of kidney allograft biopsies undergoing rejection revealed a correlation between enrichment of CD16^+^ NK cells and monocytes and severity of glomerular inflammation (17).

Whether and how NK cells function as pathological mediators in the absence of DSAs in kidney transplant injury is incompletely understood. Analyses of class I HLA and KIR genes in kidney transplant cohort studies suggest that NK cell education can influence kidney injury after transplantation. Higher numbers of mismatches between recipient KIR and donor KIR ligand–encoding HLA (missing-self) associate with a greater risk of microvascular inflammation (18, 19). The incidence of chronic rejection and graft loss is also higher with missing-self or the absence of KIR2DL1/HLA-C2 and KIR3DL1/Bw4 interactions between donor and recipient (20, 21). Beyond the observation that CD56^dim^ NK cells are elevated within allograft tissue from individuals with T cell–mediated rejection (TCMR) (22), there is limited knowledge on the functional activity of NK cells in kidney transplant recipients. Whether and how the newly recognized diversity of NK cells impacts alloimmune responses in kidney transplantation has not been carefully addressed (18). To overcome this deficiency, we performed high-dimensional cytometry by time of flight (CyTOF) on pre- and posttransplant peripheral blood mononuclear cells (PBMCs) obtained from individuals enrolled in 2 completed clinical trials and from healthy volunteers. We determined alloreactive NK cell heterogeneity following stimulation with phenotyped HLA-disparate allogeneic cells from the kidney donor and assessed how the detected phenotypic and functional subsets relate to graft function (23, 24).

## Results

### NK cell variability across healthy donors and kidney transplant recipients.

**Figure 1:**
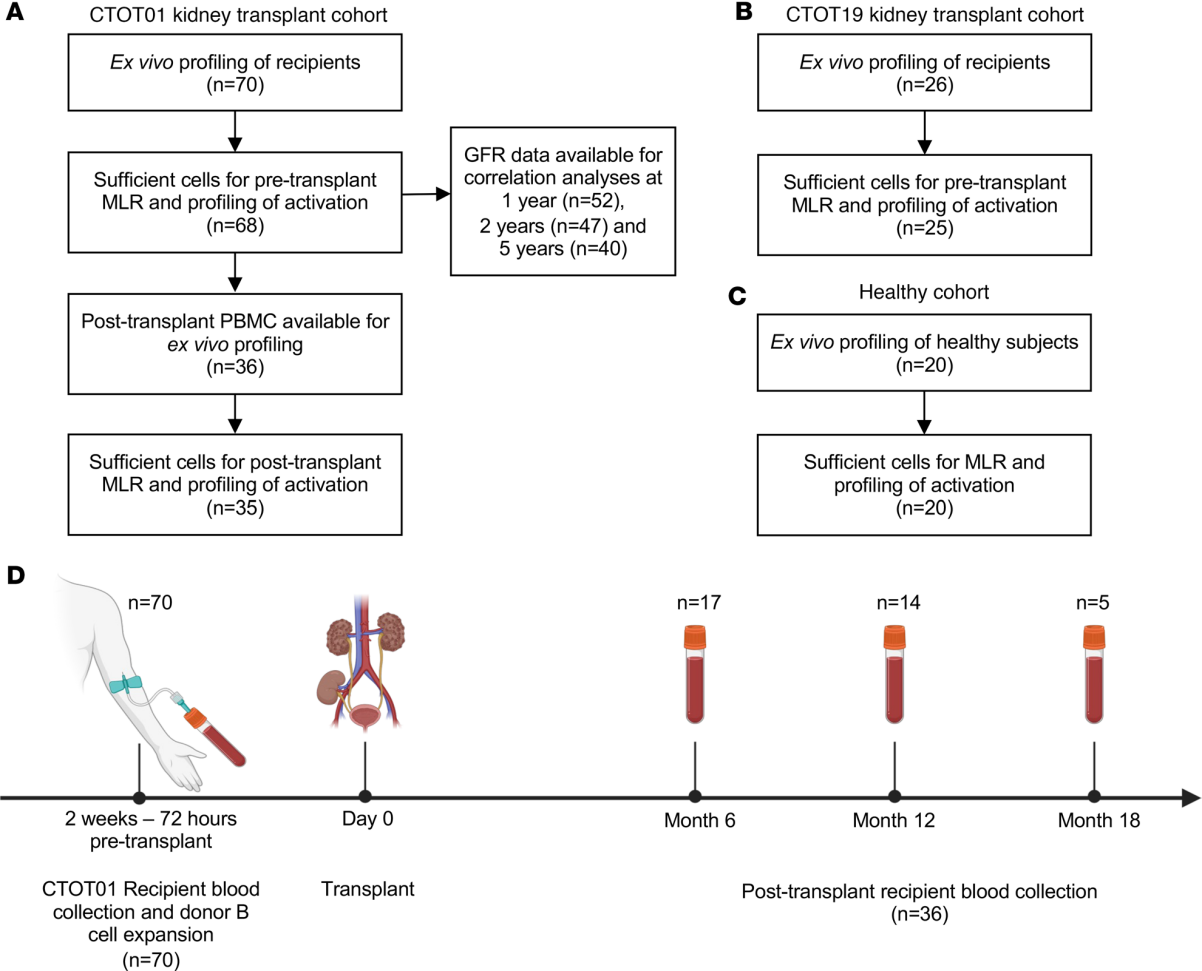
Clinical sample availability and experimental design. Consort table of (**A**) CTOT01 kidney transplant cohort (**B**) CTOT19 validation kidney transplant cohort and (**C**) healthy cohort. (**D**) Timeline of blood collection for CTOT01 cohort.

**Figure 2:**
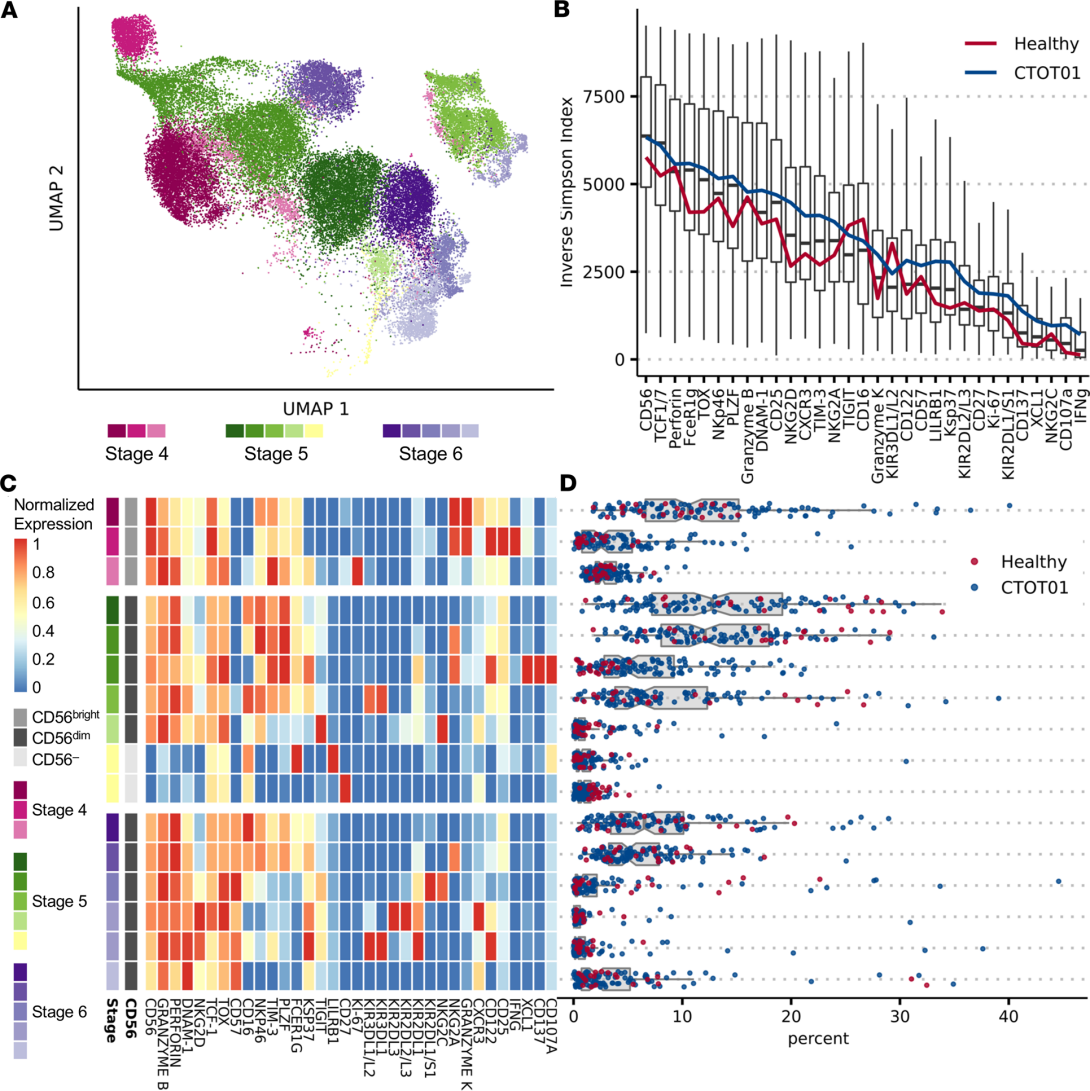
NK cells are highly diverse and vary in phenotype and composition across healthy donors and kidney transplant recipients. Cyropreserved PBMCs from healthy donor (*n* = 20) and CTOT01 kidney transplant recipients (*n* = 70) were profiled by CyTOF. (**A**) UMAP of unsupervised RPhenograph clustering of peripheral blood–derived NK cell subsets of NK cells found across stages 4, 5, and 6 of NK cell development. (**B**) Box-and-whisker plots show spread of inverse Simpson scores across healthy donors (*n* = 20) and CTOT01 participants (*n* = 70) before transplantation for each population. Lines indicate mean of inverse Simpson scores. (**C**) Heatmap shows median expression of CyTOF antibodies defining NK cell clusters. (**D**) Relative proportion of each RPhenograph cluster within NK cells of each participant.

We characterized NK cell phenotypic heterogeneity within unstimulated ex vivo PBMCs of kidney transplant recipients from the CTOT01 cohort (*n* = 70) (23) and healthy volunteers (*n* = 20) by CyTOF (Figure 1 and Table 1). We identified NK cells through manual gating of viable CD3^–^CD14^–^CD19^–^HLA-DR^dim/–^ cells that are CD56^+^CD16^+^ (Supplemental Figure 1A; supplemental material available online with this article; https://doi.org/10.1172/jci.insight.185687DS1), and distinguished underlying subsets at higher resolution through unsupervised clustering (25). In PBMCs obtained from both CTOT01 recipients and healthy volunteers, we identified 16 distinct subpopulations that reflect 3 known stages of NK cell development (26) found in the peripheral blood: stage 4 CD56^bright^ immature NK cells, stage 5 CD56^dim^ mature NK cells, and stage 6 CD56^dim^CD57^+^ terminally mature NK cells (Figure 2, A and C). Consistent with our previous report (13), the majority of NK cells fell within the CD56^dim^ subset and varied in expression of markers of education (e.g., KIRs and NKG2A), cytotoxicity (e.g., granzyme B and perforin), CD16, and the terminal maturation marker CD57 (Figure 2C and Supplemental Figure 1B). Boolean gating analysis using 31 antibodies showed that pretransplant NK cells were significantly diversified in CTOT01 recipients, with inverse Simpson scores for each NK cell receptor and functional protein ranging from a mean of 6,334 in the CD56^+^ population to a mean of 705 in the IFN-γ^+^ population (Figure 2B). CD56 expression is the most diverse given that it is expressed by most NK cells, while XCL1, CD137, CD107a, and IFN-γ expression showed the least diversity in these unstimulated, resting NK cells. While all the primary NK cell subsets were detectable across healthy volunteers and CTOT01 recipients, their relative abundance within the NK cell repertoire was highly variable across individuals (Figure 2D). Overall, we observed high variability in composition of NK cell subpopulations across CTOT01 and healthy volunteers and high heterogeneity within NK cell subpopulations. While HLA and KIR genotypes do not comprehensively predict diversity of the NK cell repertoire, genotyping of healthy individuals, kidney transplant recipients, and kidney donors was completed so that we could assess functional capacity of educated subsets.

### NK cell functional markers induced by allogeneic stimuli remain stable after transplantation.

**Figure 3:**
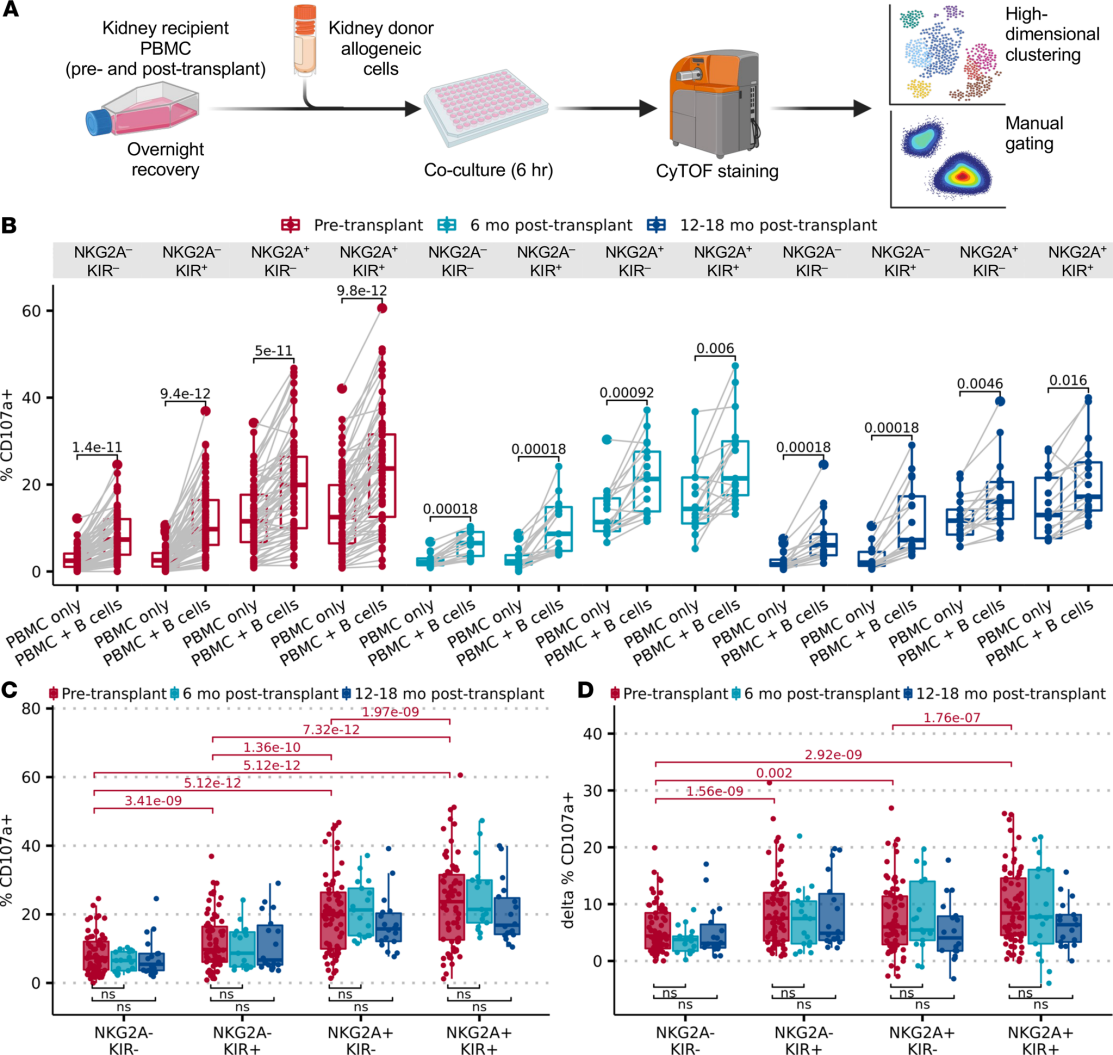
Cocultures of NK cells and allo-donor cells reveal variable alloreactivity that transcends donor differences and is maintained after transplantation. (**A**) Cryopreserved pretransplant (*n* = 70) and posttransplant (*n* = 36) PBMCs from CTOT01 kidney transplant were recovered overnight in 10 ng/mL rhIL-15 and stimulated with donor allo-stimulator B cells for 6 hours at an E/T ratio of 3:1. Results were profiled by CyTOF and subsets were defined by gating on CD56^dim^ NK cells followed by Boolean gating of educating inhibitory receptors NKG2A, KIR3DL1, KIR3DL2, KIR2DL1, and KIR2DL3. (**B**) Paired box-and-whisker plot shows change in percentage CD107a^+^ in recipient NK cells before transplantation (*n* = 70) and after transplantation (*n* = 36) with and without stimulation by donor B cells. (**C**) Box-and-whisker plot shows percentage CD107a^+^ in recipient NK cell subsets in coculture with donor cells across pre- and posttransplant time points. (**D**) Box-and-whisker plot shows B cell–dependent increase in percentage CD107a^+^ (stimulated – baseline %CD107a^+^) in recipient NK cell subsets at pre- and posttransplant time points. *P* values shown above the samples reflect Wilcoxon’s test with Bonferroni’s correction.

To assess NK cell alloreactivity, we stimulated pretransplant PBMCs from CTOT01 recipients (*n* = 70) for 6 hours with allogeneic B cell lines from their kidney donor, and quantified expression of functional markers (CD107a, IFN-γ, XCL1, Ksp37, and CD137) within each NK cell subset. We performed analogous cultures using posttransplant (6, 12, and 18 months) PBMCs from a subset of the CTOT01 kidney transplant recipients (*n* = 36) to assess functional consequences of immunosuppression and duration of transplant on NK cell alloreactivity (Figure 3A). While donor kidney cells could not be obtained for in vitro functional assays and kidney cell lines do not recapitulate the HLA allotypes of kidney donors, donor B cells were accessible and recapitulate the HLA matching between donor and recipient. Increased frequencies of donor-specific IFN-γ ELISPOTs (a measure of T cell allo-immunity) using donor B cells as stimulators correlated inversely with estimated glomerular filtration rate (eGFR) in CTOT01 kidney transplant recipients (23).

Analysis of the pretransplant samples showed that stimulation with allogeneic donor cells induced changes in expression of functional markers across CD56^dim^ NK subsets expressing either or both NKG2A and KIR. To generalize across individuals with varying KIR genetics and variable KIR expression on NK cell subsets, we defined KIR^+^ NK cells as expressing any combination of KIR3DL1, KIR3DL2, KIR2DL1, and KIR2DL3, and defined KIR^–^ NK cells as negative for all these KIRs. For the 4 subsets analyzed, NKG2A^–^KIR^–^, NKG2A^–^KIR^+^, NKG2A^+^KIR^–^, and NKG2A^+^KIR^+^ NK cells, we observed increased percentages of CD107a^+^, IFN-γ^+^, XCL1^+^, and CD137^+^ cells (Figure 3B and Supplemental Figure 2, A and B). We also analyzed expression of Ksp37, a secretory protein expressed by cytotoxic lymphocytes that has been associated with inflammatory states, including asthma and severe COVID-19 (27, 28). Intracellular staining of Ksp37, which is stored in cytoplasmic granules of resting NK cells and released upon activation, decreased when stimulated with allogeneic donor cells. Posttransplant recipient cells stimulated with donor cells also displayed increased percentages of CD107a, XCL1, and CD137. Analyses showed higher expression of total CD107a and donor-induced CD107a in subsets that express NKG2A and/or KIR (Figure 3, C and D). We observed equivalent CD107a expression before versus after transplantation at 6 months and 12–18 months despite exposure to maintenance immunosuppression at posttransplant time points. To account for functional differences between terminally mature CD57^+^ NK cells and CD57^–^ NK cells, we repeated this analysis by gating on CD57 expression in the 4 subsets (Supplemental Figure 2C). These analyses showed a similar pattern among subsets for pre- and posttransplant expression of XCL1, CD137, and IFN-γ, and higher activation in NKG2A^+^ and/or KIR^+^ NK cells. However, the NKG2A^+^ NK cells coexpressing CD57 showed higher production of IFN-γ and XCL1 than CD57^–^NKG2A^+^ NK cells, suggesting that the degree of terminal maturation affects specific effector molecules. Consistent with previous reports that found higher *FGFBP2* (gene for Ksp37) expression in more differentiated KIR^+^ NK cells (8), we observed higher intracellular Ksp37 in KIR^+^ NK subsets as well. Nonetheless, Ksp37 was still expressed across KIR^–^ subsets (Supplemental Figure 2A).

While most NKG2A^+^ and/or KIR^+^ NK cells in the CD56^dim^ subset are likely educated, the degree of education varies and is influenced by multitude combinations of educating interactions. To account for these confounders, we gated on NKG2A and specific combinations of self-KIRs relevant to the recipients’ HLA and KIR genotypes. We focused on the largest fraction of CTOT01 recipients sharing the same educating KIR ligands (Bw4^+^C1^+^C2^+^A3^–^A11^–^, *n* = 24). These recipients were educated through CD56^dim^KIR3DL1^+^ NK cells interacting with Bw4, CD56^dim^KIR2DL2/L3^+^ NK cells interacting with C1, and CD56^dim^KIR2DL1^+^ NK cells interacting with C2. Their KIR^–^ and KIR3DL2^+^ NK cells were not educated (Supplemental Figure 3A). Since these NK cells may also be educated by the inhibitory interaction between NKG2A and HLA-E, we further gated on NKG2A^+^NKG2C^–^ or NKG2A^–^NKG2C^–^ subpopulations for analysis. We tested whether the CD56^dim^ NK cells expressing all or some of the KIRs corresponding to these ligands and NKG2A responded greater to allo-stimulation compared with the uneducated CD56^dim^KIR^–^ subset (Supplemental Figure 3B). Within each subset delineated by KIR expression, we found that the CD56^dim^NKG2A^+^ compartment generally degranulated more compared with the CD56^dim^NKG2A^–^ compartment. Only 3 out of 9 subsets were not significantly different in CD56^dim^NKG2A^+^ versus CD56^dim^NKG2A^–^ after correcting for multiple comparisons. Within the CD56^dim^NKG2A^–^ subsets, all CD56^dim^KIR^+^ subsets, regardless of the number of KIRs, were more activated than the uneducated CD56^dim^KIR^–^ cells. Interestingly, even the CD56^dim^KIR3DL2 single-positive subset was more activated than the KIR^–^ cells despite lacking the educating A3 or A11 ligands in these recipients.

To determine the effects of missing-self on Bw4-educated KIR3DL1^+^ NK cells, we categorized donor/recipient pairs based on copies of Bw4 encoded by their *HLA-A* and *HLA-B* alleles, testing whether lower Bw4 copy number influences responsiveness. We categorized Bw4^+^ recipients paired with donors with fewer or no copies of Bw4 as loss of Bw4 (*n* = 17). Recipients without loss of Bw4 had donors with equal or greater copy number of Bw4 or do not express KIR3DL1 (*n* = 48). Within the pretransplant KIR3DL1^+^ NK subset, we found that those with Bw4 loss expressed more IFN-γ, XCL1, and CD107a compared with those without loss of Bw4 (Supplemental Figure 3C). We also observed a significant reduction in Ksp37 staining in the KIR3DL1^+^ NK subset with Bw4 loss compared with recipients without Bw4 loss, suggesting that reduced Bw4 inhibition of KIR3DL1^+^ NK cells promotes Ksp37 release.

Independent of education, activating interactions (29) can modulate the degree of alloreactive NK cell effector function. We performed cocultures in the presence/absence of blocking antibodies against the NKG2D and DNAM-1 activating receptors in healthy volunteer PBMCs. NK cell activation induced by missing-self and/or non-self was abrogated by blocking NKG2D and DNAM-1 (Supplemental Figure 4A). The reduction in CD107a degranulation by NKG2D^+^DNAM-1^+^ blockade was greatest when the NK cells were cocultured with wild-type and HLA-E^+^ K562, which highly expressed ligands for NKG2D and DNAM-1. Since the percentages of NKG2D^+^ and DNAM-1^+^ NK cells in the CTOT01 transplant cohort before and after transplantation were higher than in the healthy cohort ex vivo, their NK cell alloreactive response may be further abrogated by NKG2D and DNAM-1 blockade (Supplemental Figure 4B).

### Pretransplant NK cell antidonor reactivity correlates with late allograft function.

Since missing-self as determined by HLA/KIR genotyping independently associates with microvascular injury (MVI) and similar numbers of NK cells have been detected in MVI^+^DSA^+^C3d^−^ and MVI^+^DSA^–^ biopsies (19, 30), we tested the hypothesis that pretransplant NK cell alloreactivity correlates with posttransplantation antibody-independent kidney allograft function. We used eGFR as the endpoint for these analyses because (i) eGFR is a reliable surrogate endpoint for kidney disease progression in native kidneys and is commonly used in kidney transplant trials (31, 32), (ii) eGFR is a crucial component of the iBox predictive model being assessed by the FDA as a clinical trial endpoint (33, 34), and (iii) in previous work using the CTOT01 cohort we showed that eGFR analyses 2 years after transplantation identified individuals with an elevated risk of graft loss (35).

**Figure 4:**
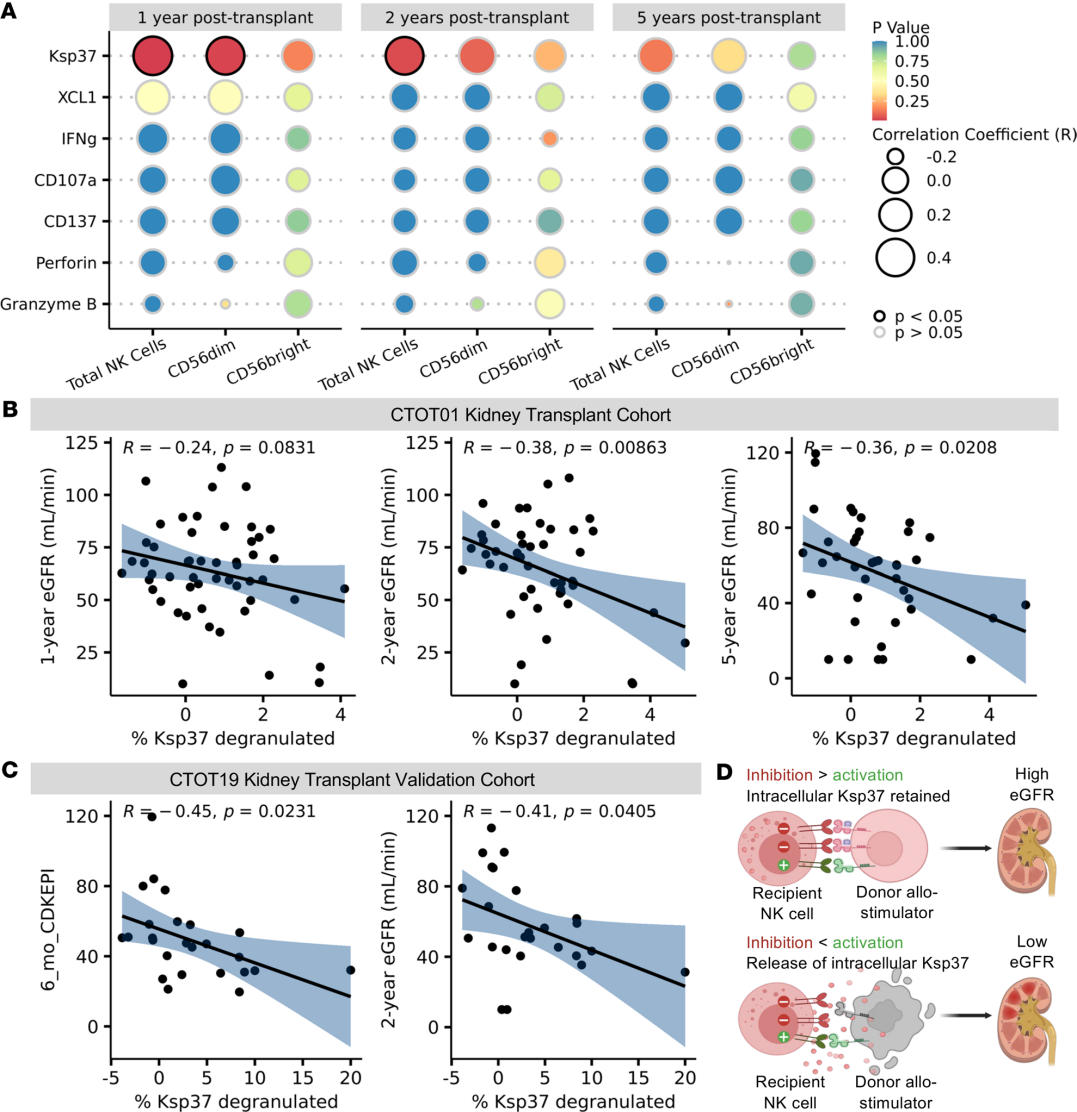
Increased Ksp37 degranulation in NK cells inversely correlates with lower estimated glomerular filtration rate (eGFR). (**A**) Bubble plot of correlations between change in percentage of effector molecule–positive cells among NK cells after allo-donor stimulation (percentage in B cell coculture – percentage in baseline) and eGFR at 1 year (*n* = 52), 2 years (*n* = 47), and 5 years (*n* = 40) in CTOT01. (**B**) Percentage Ksp37 degranulated calculated as percentage Ksp37^+^ cells among total NK cells in B cell coculture condition minus PBMC-only condition. eGFR values were calculated with CKD-EPI formula and patients with graft failure were assigned eGFR = 10 mL/min. Scatter plots show inverse correlation of Ksp37 degranulation in CD57^–^NKG2A^+^KIR^–^ NK cells with eGFR. (**C**) Scatter plots show inverse correlation of Ksp37 degranulation in CD57^–^NKG2A^+^KIR^–^ NK cells and 6-month and 2-year eGFR (*n* = 25) in CTOT19 validation cohort. (**D**) Model of Ksp37 release induced by allogeneic donor cell leading to reduced kidney allograft function. Correlation coefficient and *P* value shown for Pearson’s correlation.

We specifically tested the hypothesis that a granular analysis of specific subsets and the strength of their responses to allogeneic donor cells, rather than cell type abundance, would correlate with late allograft function. We tested for associations of donor-induced changes in CD107a, IFN-γ, CD137, XCL1, granzyme B, perforin, and Ksp37 within total NK cells and subsets of CD56^bright^ and CD56^dim^ NK cells, with eGFR at 1, 2, and 5 years after transplantation (Figure 4A). These analyses uncovered that donor-induced Ksp37 release inversely correlated with eGFR at 1–5 years after transplantation. To account for the impact of maturation on alloreactivity, we gated on total NK cells based on expression of the CD57 terminal maturation marker. To account for the effects of education, we gated on CD57^+^ and CD57^–^ NK cell subsets based on NKG2A and KIR expression. Boolean gating on CD57, NKG2A, and KIR resulted in 8 NK cell subsets. We tested the strength of the association between the change in Ksp37^+^ frequencies of each subset with eGFR. This analysis identified that only loss of intracellular Ksp37 within the CD57^–^NKG2A^+^KIR^–^ subset significantly (and inversely) correlated with eGFR at 2 years and 5 years after transplantation (Figure 4B). The inverse correlation of Ksp37 degranulation and 2- to 5-year eGFR remained significant after correcting for induction thymoglobulin, rejection status, living/deceased status of transplant donor, degree of HLA mismatch, donor/recipient age, and donor/recipient sex across multiple multivariate linear regression models (Supplemental Table 1). We did not correct for delayed graft function (DGF), as DGF occurred in only 3% (2 out of 69) in the discovery CTOT01 cohort (36), which consisted of 70% living donor transplants (Table 1). This range is comparable to previously reported rates of DGF in other living donor cohorts (37). This relationship between Ksp37 response in CD57^–^NKG2A^+^KIR^–^ NK cells and eGFR was unique among other functional markers (IFN-γ, CD107a, XCL1, and CD137), which did not correlate with 1-, 2-, or 5-year eGFR.

Notably, we did not detect associations between the relative sizes of donor-stimulated CD56^bright^ or CD56^dim^ subsets and 1-, 2-, or 5-year eGFR (Supplemental Figure 5). Nor did we find associations between eGFR and percentage of other functional markers on NK cells, indicating that only Ksp37 produced by the CD57^–^NKG2A^+^KIR^–^ subset is informative. We also checked for associations between eGFR and the subpopulations of NK cells defined by unsupervised clustering (Supplemental Figure 6A). The percentage of CD57^+^NKG2A^+^ NK cells nominally correlated with 5-year eGFR, but this association was not significant after correcting for multiple comparisons.

As validation, we used PBMCs from donor/recipient pairs from the distinct, deceased donor, kidney transplant CTOT19 cohort (24) (Table 1). These assays similarly showed a significant decrease in percentage of Ksp37^+^ cells among CD57^–^NKG2A^+^KIR^–^ NK cells that correlated inversely with 6-month and 2-year eGFR (*n* = 25, Figure 4, C and D). The association between Ksp37 degranulation and eGFR was significant after correcting for delayed graft function and recipient sex at 6 months and 2 years (Supplemental Table 2). The association was also independent of acute rejection and number of HLA-A/B/C mismatches at 6 months (Supplemental Table 2). The DSA rates were 4.3% (3 out of 69) and 3.8% (1 out of 26) for CTOT01 and CTOT19, respectively (Table 1), which are similar to reported DSA rates detected within 1–5 posttransplant years (mean ~5%) (38, 39). Due to an insufficient number of recipients with detectable DSAs, we could not correct for the effect of DSAs in our multivariate models. We did not observe a significant correlation between change in Ksp37^+^ cells among CD57^–^NKG2A^+^KIR^–^ NK cells and change in eGFR between 6 months and 24 months in our cohort, consistent with previous reports that showed minimal change between eGFR at these 2 time points (40).

### Allo-induced Ksp37 release correlates with NK cell cytotoxicity.

**Figure 5:**
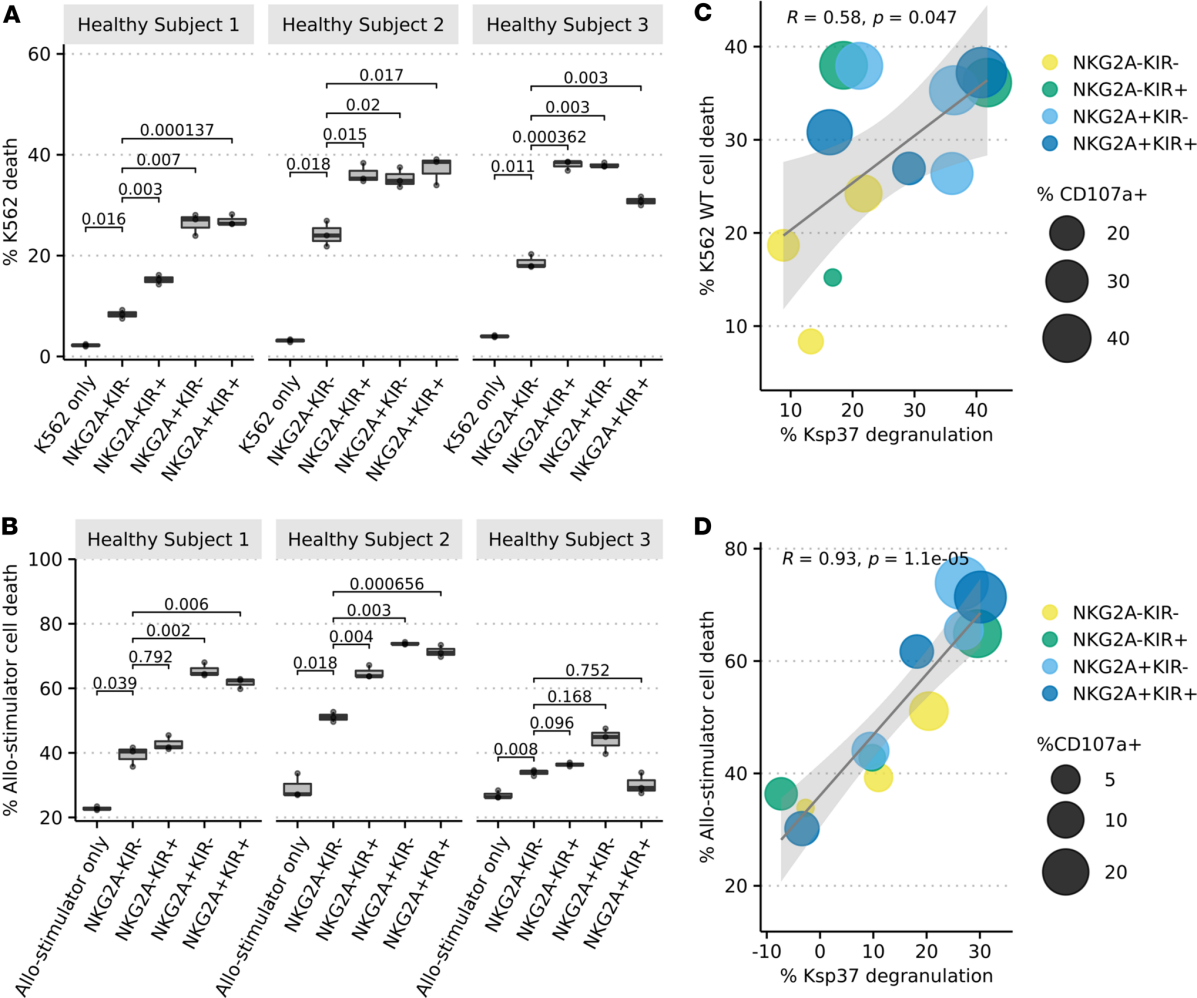
NK cell subsets enriched for education are more potent allo-reactive killers. Flow-sorted NK cells from healthy donors were cocultured with K562 and allo-stimulator B cells at an E/T ratio of 1:1 for 5 hours to assess NK cell killing and activation. Box-and-whisker plots show (**A**) K562 cell death and (**B**) allo-stimulator cell death at baseline and with addition of purified NK cell subsets in culture across 3 technical replicates. (**C**) Correlation of NK cell Ksp37 release with percentage of K562 death and (**D**) allo-stimulator cell death. *P* values in **A** and **B** were calculated using a 2-tailed Student’s *t* test with Bonferroni’s correction. *P* values in **C** and **D** were calculated by Pearson’s correlation.

To investigate activated NK cell killing potential, we flow sorted NKG2A^–^KIR^–^, NKG2A^–^KIR^+^, NKG2A^+^KIR^–^, and NKG2A^+^KIR^+^ NK cells from 3 healthy donors (KIR^+^ defined as staining positively for anti-KIR3DL1/L2 or KIR2D). To avoid confounding effects of the activating NKG2C/HLA-E interaction, we excluded all NKG2C^+^ cells from these comparisons. We stimulated the NK cell populations with K562 and allo-stimulator cells and analyzed stimulator cell death and NK cell CD107a and Ksp37 expression by flow cytometry. These assays showed significant NK cell–induced death of K562 and allo-stimulators, with greater killing by the NKG2A^+^ than NKG2A^–^KIR^–^ NK cells despite heterogeneity among the individuals (Figure 5, A and B). Across these NK subsets, the degree of K562 and allo-stimulator cell death correlated with lower intracellular Ksp37 (implying release induced by activation) and higher CD107a expression (Figure 5, C and D). The majority of Ksp37 loss, CD107a expression, and stimulator cell death was dominantly mediated by the NK cell subsets that express NKG2A and/or KIR. Collectively, our data suggest Ksp37 functions as a sensitive marker of educated NKG2A^+^ and KIR^+^ NK cell activation/cytotoxicity in response to allogeneic stimuli.

### Intragraft FGFBP2 gene (Ksp37) expression correlates with late graft loss.

**Figure 6:**
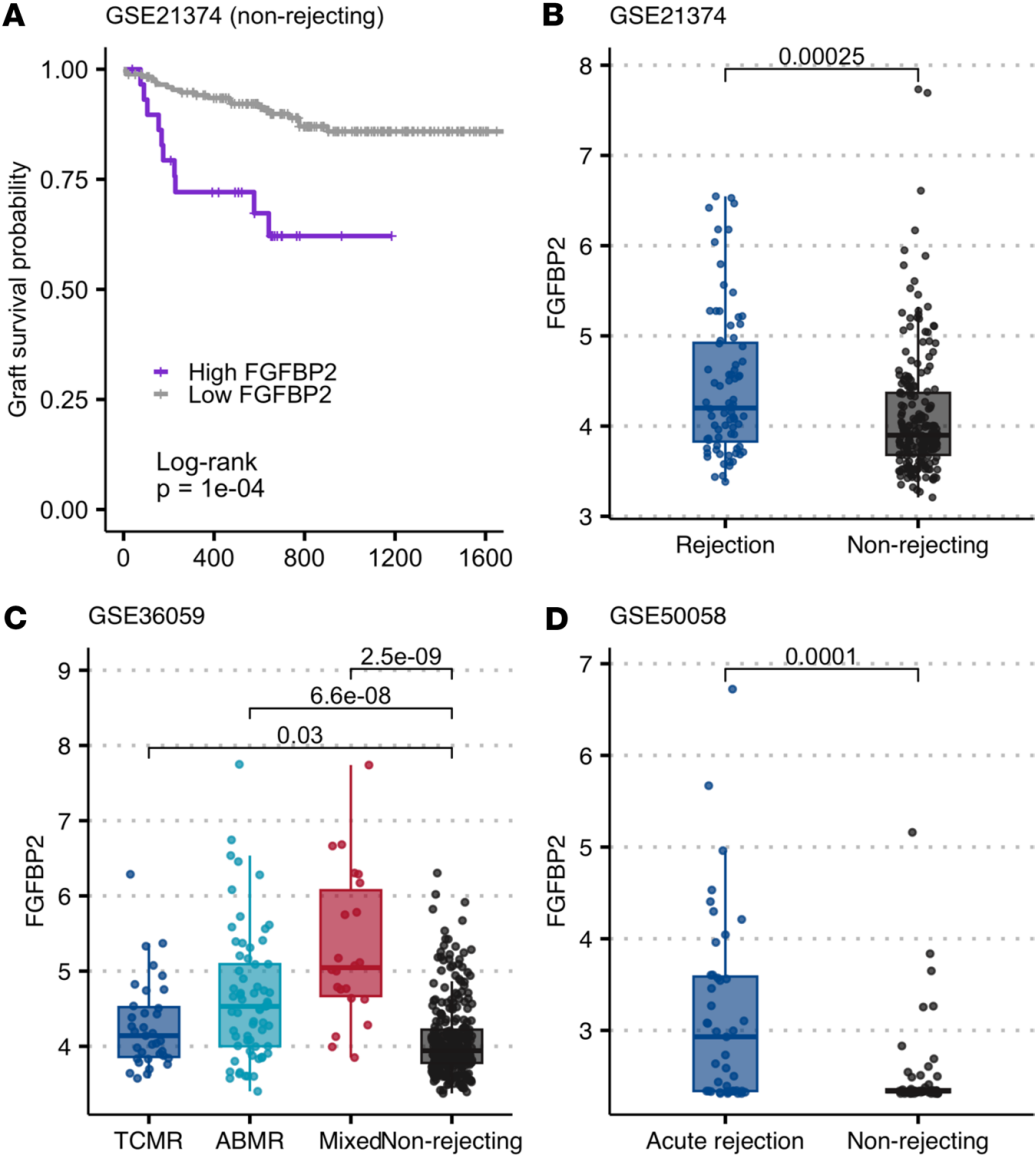
Increased expression of *FGFBP2* in allograft rejection and graft failure. Microarray gene expression of *FGFBP2* was extracted from GEO GSE36059, GSE50058, and GSE21374. (**A**) Kaplan-Meier plot shows time to graft failure in subset of non-rejecting recipients from GSE21374 with either high (*n* = 30) or low (*n* = 176) expression of *FGFBP2*. High and low expression of *FGFBP2* was determined by StepMiner, as previously reported (43). (**B**) *FGFBP2* expression in GSE21374 was higher in histologically defined rejection (*n* = 76) than non-rejection (*n* = 206). (**C**) *FGFBP2* expression in GSE36059 was higher in histologically defined TCMR (*n* = 35), ABMR (*n* = 65), and mixed rejection (*n* = 22) than non-rejection (*n* = 289). (**D**) *FGFBP2* expression in GSE50058 was higher in histologically defined acute rejection (*n* = 43) than non-rejection (*n* = 58). *P* value in **A** was calculated by log-rank test; *P* values in **B**–**D** reflect Wilcoxon’s test with Bonferroni’s correction.

As an independent validation strategy, we mined previously published data from 3 cohorts of kidney transplant recipients in which for-cause allograft biopsies were subjected to microarray-based transcriptomics and deposited into the NCBI Gene Expression Omnibus (GEO) database (41–43). Analyses of GSE21374 transcriptional data from the subset of allografts lacking clinical/histological evidence of rejection over 31 years remarkably showed significantly higher probability of graft loss in recipients expressing higher levels of *FGFBP2* (gene for Ksp37) (Figure 6A). *FGFBP2* expression level was classified as high or low by StepMiner, a computational tool that uses an adaptive regression method to reduce square error (44). The same analysis was repeated for *IFNG* and *LAMP1* (gene for CD107a), but these genes did not associate with increased probability of graft loss in patients without histological evidence of rejection (Supplemental Figure 6, B and C). Cox regression analysis revealed that the effect of *FGFBP2* is independent of *IFNG* and *LAMP1* expression (Supplemental Figure 6, D and E). In analyses that included biopsies with histological evidence of rejection (T cell–mediated, antibody-mediated, or mixed), intragraft expression levels of *FGFBP2* were significantly higher in rejection compared with non-rejection in all 3 microarray datasets (Figure 6, B–D).

## Discussion

Although NK cells play critical roles in controlling cancers and microbial infections, their roles in the transplantation of solid organs lacks a nuanced understanding. Previous studies of ABMR demonstrated that NK cells and KIR genetics associate with kidney transplant outcomes independent of T cells and DSAs and have suggested ADCC as the mechanism by which NK cells contribute to graft injury (17, 45, 46). Where DSAs often reflect allo-HLA antibodies, one such alternate trigger is the class I HLA allotype itself. If donor tissue lacks KIR ligands from class I HLA that are present in the recipient, then the recipient’s NK cells may react against the graft, a process referred to as “non-self” activation. Understanding the requirement to maintain these inhibitory interactions requires the study of class I HLA, KIR, and NKG2A immunogenetics that inform NK cell education (18, 19, 22, 30). Here, we analyzed HLA/KIR/NKG2A immunogenetics in donors and recipients alongside high-dimensional profiling of recipient-derived NK cell subsets and donor-derived cells to elucidate the mechanisms by which variably educated NK cell subsets contribute to long-term graft function.

CyTOF analyses of blood-derived NK cells revealed a high degree of heterogeneity where composition of NK cells varied between kidney transplant recipients and diversity of functional receptors and effector molecules varied across subsets. Within these subsets, we analyzed their responses to allogeneic donor cells, which also varied in the expression of ligands that modulate NK cell response. We hypothesized that particular subsets, for example, the NKG2A^+^ subsets that are strongly educated through HLA-E, may be major contributors to NK cell alloreactivity in kidney transplantation. NKG2A is a well-established inhibitory receptor expressed by subsets of NK cells and CD8^+^ T cells that interacts with the non-classical HLA-E molecule (3). During NK cell development, HLA-E trains NKG2A^+^ NK cells to distinguish healthy cells with normal expression of HLA-E from diseased cells, which may exhibit perturbed expression of HLA-E. In allogeneic hematopoietic cell transplantation, increased numbers of NKG2A^+^ NK cells have been shown to predict graft-versus-host disease (47, 48). Additionally, previous reports have found that a higher frequency of NKG2A^+^ NK cells relative to NKG2D^+^ NK cells associates with lower eGFR (49, 50). In support of these findings, the CD57^–^NKG2A^+^KIR^–^ population in our transplant cohorts released Ksp37 that associated with poor long-term graft function and offers an additional pathogenic role for NKG2A^+^ NK cells in kidney functions following transplantation.

Studies of related fibroblast growth factor–binding (FGF-binding) proteins, FGBP1 and FBFB3, suggest that *FGFBP2* (gene encoding Ksp37) is released by cells to bind to heparin and FGF ligands to aid the binding of FGF-2 to FGFR1 (51–53). Intracellular staining of Ksp37 and perforin in NK and T cells revealed that these molecules are stored in discrete granules, suggesting that the mechanism of regulation of Ksp37 is likely distinct from that of cytotoxic molecules like perforin and granzymes (54). While the function and regulation of Ksp37 in immune cells is not clear, there have been multiple studies linking it to cytotoxic cell immunity in settings of infection and inflammation. Ksp37^+^ T cells are increased in patients with asthma (27), Ksp37^+^CD56^bright^ NK cells are increased in patients with severe COVID-19 (28), and Ksp37 serum levels are increased in early EBV infection (55). Similar to these pathologies, Ksp37 may be an indicator of NK cell activation in allograft dysfunction. Although *FGFBP2* has been incorporated as part of gene modules that associate with ABMR (56), this is the first demonstration to our knowledge of *FGFBP2* as a predictor of graft loss independent of histologic rejection. We acknowledge that molecular evidence of ABMR can be detected in allografts that lack pathological evidence of ABMR, even in the absence of serum DSAs (16, 57). Consequently, while our analysis showing allograft *FGFBP2* expression in the absence of rejection correlates with late graft loss supports the conclusion that NK cells contribute to antibody-independent graft injury, additional confirmatory studies will be required.

The donor-induced release of Ksp37 in our in vitro models also offers a mechanism to explain the detection of this transcript in prior studies of gene signatures of chronic injury in kidney transplantation (14, 16, 58). Since our model for predicting eGFR is independent of rejection, it bolsters emerging evidence that NK cells contribute to graft injury regardless of rejection status (TCMR or ABMR). In an independent microarray dataset, we also showed that Ksp37 transcripts associated with greater graft failure even in patients without histologic rejection. In particular, it might explain why a higher degree of missing/non-self, as determined by recipient and donor genotype, has been associated with MVI, which does not meet the BANFF criteria for rejection (30).

NK cell education is preserved and engaged in kidney transplant patients, and the magnitude of alloreactivity transcends differences among kidney donor cells. The increase in XCL1, IFN-γ, and CD107a, and decrease in Ksp37 in KIR3DL1^+^ NK cells stimulated by allo-donor cells deficient in or presenting fewer copies of Bw4 provide a mechanism for the genetic association studies linking the absence of recipient KIR3DL1 and donor Bw4, and other KIR ligand mismatches, to increased incidence of chronic rejection (20, 21).

We also showed that the effects of education on alloreactivity persist after transplantation after NK cells have been exposed to induction immunosuppression and continued exposure to maintenance immunosuppression. The 2 most common types of induction immunosuppression, rabbit anti-thymocyte globulin (rATG) and blocking anti–IL-2 receptor α (anti-IL2RA) antibodies, deplete T cells (rATG), or reduce T cell proliferation (IL2RA) and have demonstrated efficacy in lowering early acute rejection (59). Maintenance immunosuppression following kidney transplantation commonly consists of calcineurin inhibitors such as cyclosporin A and tacrolimus, mTOR inhibitors, and mycophenolate, which have been shown to reduce rejection by reducing T cell proliferation and activation. In vitro ADCC assays on peripheral blood–derived NK cells performed by others showed that cyclosporine A and tacrolimus reduce IFN-γ and CD107a at high doses, while mycophenolate and sirolimus have minimal effects (60). Their effects on NK alloreactivity in kidney transplantation is less well understood. Recent studies characterizing the effects of mTOR and calcineurin inhibitors on NK cell functions in solid organ transplantation presented discordant results, with some evidence suggesting that (i) NK cells from kidney transplant recipients vary expression of activation markers depending on the type of immunosuppression administered and (ii) that posttransplant NK cells from calcineurin-treated recipients produce lower levels of cytokines compared with healthy individuals (61, 62). Yet, other studies show in murine models of kidney transplantation that calcineurin inhibitors fail to prevent NK cell–mediated rejection, while mTOR inhibitors may reduce NK cell missing-self–induced MVI (30, 63).

Our results showed greater alloreactivity by NKG2A^+^KIR^+^ NK cells versus uneducated NKG2A^–^KIR^–^ NK cells and that this effect was maintained after transplantation. The data indicated that maintenance immunosuppression does not impede this facet of alloreactivity. Although we observed a small decrease in the abundance of posttransplant circulating IFN-γ^+^ and XCL1^+^ NK cells compared with pretransplant, it was restricted to the non-educated NKG2A^–^KIR^–^ NK cells, which have lowered alloreactivity in general.

Our data also show that in vitro NK cell alloreactivity is reduced by activating receptor (NKG2D and DNAM-1) blockade. In murine models, NKG2D expression increases over the course of ischemic injury and this injury is reduced by the adoptive transfer of *NKG2D*^–/–^ NK cells or through blockade of NKG2D (64). Similarly, NKG2D blockade reduces cardiac vasculopathy in murine heart transplantation (65, 66). Our CyTOF analyses of circulating NK cells showed that NKG2D and DNAM-1 are highly expressed in kidney transplant recipients before and after transplantation; thus, there is potential for targeting these 2 activating receptors, among other activating pathways, to reduce kidney injury. NKG2C is another key activating receptor that regulates NK cell activity. It is primarily expressed on adaptive NK cells that are expanded from cytomegalovirus (CMV) infection and plays a role in controlling CMV viremia in kidney transplant recipients (67, 68). Due to the limited number of patients (5 out of 69) with expansions of adaptive NKG2C^+^ NK cells in our discovery cohort, we could not address the alloreactive potential of this subset, although one study has pointed to the possibility of *KLRC2* (gene encoding NKG2C) associating with MVI in DSA^+^ kidney transplant recipients (69).

Future studies to visualize NK cell subsets localized to areas of immune infiltrate or fibrosis may clarify the helper or direct roles of NK cells in mediating graft injury. The results of the current study provide an in-depth analysis of the heterogeneity of NK cells found in the periphery of transplant recipients and how education and expression of inhibitory/activating receptors guide their alloreactive response. We provide an explanation for the published associations between genotype and outcomes and raise the possibility that pretransplant NK cell analyses could be used as risk assessment biomarkers for kidney transplantation.

## Methods

### Sex as a biological variable.

Our study included male and female participants and sex was included as a covariate in linear regression models to predict outcome.

### Cohorts and participants.

CTOT01 was a prospective multicenter observational trial of kidney transplants from 2006 to 2009. Trial design and details on participants have been previously published (23). CTOT19 was a randomized control trial to test infliximab as induction therapy in deceased-donor kidney transplantation from 2016 to 2021. Trial design and details on participants have been previously published (24). The CTOT01 (*n* = 70) and CTOT19 (*n* = 26) participants studied herein were chosen based on sample availability. Values for eGFR were extracted from the primary publications. CTOT01 2-year and 5-year eGFR values were calculated with the Chronic Kidney Disease Epidemiology Collaboration (CKD-EPI) formula for adults and the Schwartz formula for children (23, 35). CTOT19 6-month and 24-month eGFR values used in this study were calculated with the CKD-EPI equation (24). Patients with graft failure were assigned an eGFR of 10 mL/min. PBMCs from the healthy cohort (*n* = 20) were sourced as buffy coats from the New York Blood Center and from the Bhardwaj lab (Mount Sinai, New York, New York, USA).

### HLA and KIR typing.

DNA was extracted from frozen whole blood, buffy coats, cryopreserved PBMCs, and cryopreserved B cells using the DNeasy Blood & Tissue Kit (Qiagen, 69506). Class I HLA and KIR typing was performed in-house and by CD Genomics.

### Profiling stimulator cell lines.

Allo-stimulator B cells were obtained from a previous study where transplant donor primary B cells were expanded using CD40L-transfected fibroblasts and IL-4, according to a previously published protocol (23, 70). K562 cell lines were provided by Deepta Bhattacharya (University of Arizona, Tucson, Arizona, USA). All stimulator cells were profiled for activating and inhibitory ligands by staining with a viability dye and surface antibodies in FACS buffer (Dulbecco’s PBS [DPBS], 2% heat-inactivated FBS, 2 mM EDTA) for 30 minutes on ice (Supplemental Table 3); cells were stained in triplicate with isotype controls. After washing, cells were fixed with 2% paraformaldehyde (PFA) (EMS, 15710) for 10 minutes at room temperature, resuspended in FACS buffer, and stored at 4°C until data acquisition on an LSRFortessa (BD Biosciences). Results were analyzed using Cytobank software (Beckman Coulter) and R software version 4.0.3 (https://www.r-project.org/).

### In vitro cocultures.

Transplant recipient and healthy donor cryopreserved PBMCs were thawed and recovered overnight in 10 ng/mL recombinant human IL-15 (Peprotech, 200-15) at 2 × 10^6^ cells/mL cell culture media (RPMI-1640, 10% heat-inactivated FBS, 1% penicillin, 1% streptomycin, 1% L-glutamine). Allo-stimulator B cells were thawed immediately prior to coculture. After overnight recovery, PBMCs were cocultured with donor allo-stimulator B cells, K562 wild-type, or K562 HLA-E^+^ cell lines at an effector/target (E/T) ratio of 3:1 for 6 hours in cell culture media supplemented with anti-CD107a-172Yb (Miltenyi Biotec, 130-124-536) in 96-well round-bottom plates. Plates were centrifuged at 100*g* for 2 minutes at start of coculture. Following 1 hour of incubation, Brefeldin (BioLegend, 420601) and Monensin (BioLegend, 420701) were added for the remainder of coculture. For NKG2D/DNAM-1 blockade in the healthy cohort, PBMCs were incubated with a cocktail of anti-NKG2D-145Nd (BioLegend, 320814) and anti–DNAM-1-146Nd (Life Technologies, MA5-28149) or mouse IgG1 κ isotype control (BioLegend, 401402) for 30 minutes at room temperature prior to addition of stimulator cells. Assays were terminated by placing plates on ice for 10 minutes and proceeding to the CyTOF staining protocol.

### CyTOF staining.

Antibodies were either purchased in metal-conjugated form from Standard BioTools or purchased as purified carrier-free antibodies and conjugated following Standard BioTools’ Maxpar Labelling protocols (Supplemental Table 3). Antibodies were titrated, and master mixes of cocktails were prepared prior to experiments. Technical replicates from cocultures were pooled and washed in cell staining media (PBS, 0.2% BSA). Samples were Fc-blocked (BioLegend, 422302) on ice for 5 minutes and stained with anti-β2m Platinum (Pt) barcodes (Human Immune Monitoring Core, Mount Sinai) for 30 minutes on ice. 194Pt-, 195Pt-, and 196Pt-barcoded samples were pooled for surface staining with antibody cocktail and Rh103 (Standard BioTools, 201103A) for 30 minutes at room temperature. Surface antibody cocktail for healthy cohort samples blocked with anti-NKG2D-145Nd and anti-DNAM-1-146Nd in culture excluded the staining antibodies for anti-NKG2D and anti–DNAM-1. After surface staining, CTOT01 and healthy donor cocultures were fixed and permeabilized following the manufacturer’s protocol using the eBioscience Foxp3/Transcription Factor Staining Buffer Set (Thermo Fisher Scientific, 00-5523-00). For CTOT19 coculture, cells were fixed and permeabilized following the manufacturer’s protocol using the Cytofix/Cytoperm Fixation/Permeabilization Kit (BD Biosciences, 554714). After centrifugation and washing, samples were stained with a Cell-ID 20-Plex Pd Barcoding kit (Standard BioTools, 201060) for 30 minutes at room temperature. Barcoded samples were then pooled for intracellular staining for 30 minutes on ice with antibody cocktail supplemented with 100 U/mL heparin. After washing, cells were fixed and Ir-stained with 2.4% PFA, 1% saponin, and 0.05% Ir (Standard BioTools, 201192A). Samples of all pooled cells were stored at –80°C in 10% DMSO/FBS until acquisition.

### CyTOF analyses.

Live, intact NK cells were identified by gating in CytoBank, and events were exported for analysis in R software version 4.0.3. A maximum of 2000 events from each participant/experimental condition were randomly selected for all downstream unsupervised clustering analyses. All events for live, intact NK cells were included for manual gating analyses. Preprocessing of FCS files and events for analysis was done with cytoqc version 0.99.2 (https://github.com/RGLab/cytoqc) and MetaCyto version 1.12.0 (https://www.bioconductor.org/packages/release/bioc/html/MetaCyto.html). Expression values of each marker were arcsinh transformed with a cofactor of 5. Unsupervised clustering was performed with R package Rphenoannoy version 0.1.0, and dimensionality reduction for generating UMAPs was done with R package umap version 0.2.10.0. Additional manual gating and Boolean gating to define NK subpopulations were done using flowCore version 2.2.0 (https://bioconductor.org/packages/release/bioc/html/flowCore.html) and premessa version 0.3.2 (https://github.com/ParkerICI/premessa). Hierarchical clustering and visualization of heatmaps were generated with pheatmap version 1.0.12 (https://github.com/raivokolde/pheatmap).

### NK cell killing assay and flow cytometry analyses.

PBMCs from healthy donor buffy coats were isolated by Ficoll density centrifugation and rested overnight in 10 ng/mL recombinant human IL-15 (Peprotech, 200-15) at 2 × 10^6^ cells/mL cell culture media (RPMI-1640, 10% FBS, 1% penicillin, 1% streptomycin, and 1% L-glutamine). After overnight recovery, PBMCs were enriched for NK cells by negative selection following the manufacturer’s protocol (Stemcell Technologies, 19055) and surfaced stained in FACS buffer (DPBS, 5% heat-inactivated FBS, 2 mM EDTA) with a cocktail of antibodies against CD3, CD56, NKG2A, NKG2C, KIR3DL1/L2, and KIR2D for 30 minutes on ice (Supplemental Table 3). Stained cells were washed and resuspended in sorting buffer (RPMI-1640, 2% heat-inactivated FBS, 1% penicillin, 1% streptomycin, 1% L-glutamine, 1:3000 propidium iodide) prior to sorting on a Cytoflex SRT (Beckman Coulter Life Sciences). Stimulator cells were stained with CFSE (1:33,000) following the manufacturer’s protocol (Life Technologies, C34570). Sorted NK subsets were cocultured with CFSE-stained stimulator cells at an E/T ratio of 1:1 in cell culture media supplemented with anti-CD107a in 96-well V-bottom plates. Plates were centrifuged at 100*g* for 2 minutes at start of coculture. After 5 hours, assay was ended by placing plate on ice for 10 minutes before proceeding to viability staining with Zombie NIR and surface staining as detailed above. Surface-stained cells were fixed with 2% PFA for 10 minutes at room temperature, permeabilized (BioLegend, 421002) for 30 minutes on ice, and stained with intracellular anti-Ksp37 for 30 minutes on ice. Cells were fixed with 2% PFA for 10 minutes at room temperature, resuspended in FACS buffer, and stored at 4°C until acquisition on an LSRFortessa (BD Biosciences). Results were analyzed using Cytobank and R software version 4.0.3.

### Microarray datasets.

Microarray profiling of kidney allografts from transplant recipients undergoing biopsies for cause were obtained from GSE36059, GSE50058, and GSE21374 through the NCBI GEO database. High and low expression of *FGFBP2*, *IFNG*, and *LAMP1* was determined using the StepMiner computational tool (44).

### Illustrations and graphs.

Figure 1D, Figure 3A, Figure 4D, and Supplemental Figure 3A were created with BioRender.com. Graphs were generated with GraphPad Prism or R package ggplot2 version 3.3.6.

### Statistics.

Statistical tests are indicated in figure legends. A *P* value of less than 0.05 was considered significant. Statistical analyses were performed using R software version 4.0.3 and GraphPad Prism software version 9.5.1. R packages used for statistical tests include rstatix version 0.7.2, ggpubr version 0.4.0, stats version 4.0.3, survival version 3.5.5, and survminer 0.4.9. Living/deceased status of transplant donor, HLA-A/B-DR mismatch, age, and sex were included as covariates in regression models based on univariate analyses where *P* was less than 0.1. Additional covariates, including thymoglobulin, acute rejection, and delayed graft function, were added based on prior studies that have shown correlations with outcome. To prevent overfitting, multiple iterations of regression models were used to test all covariates that could be accommodated by size of cohorts. Box-and-whisker plots show the median and 95% interquartile range.

### Data availability.

Values for all data points in box-and-whisker plots and scatter plots are reported in the Supporting Data Values file. Additional data from this study are available from corresponding authors upon request.

## Author contributions

PSH and AH provided study oversight and obtained funding. DFR performed and analyzed experiments, prepared figures, and wrote the initial draft of the manuscript. PSH and AH edited and wrote the manuscript. MF, NC, and PC helped with conceptualization of the project. YY, MPM, and MC provided support for HLA/KIR typing. HY provided guidance on navigating the GEO database. GCK, BL, RMR, RL, DG, and SKS helped with data acquisition.

## Supplementary Material

Supplemental data

Supporting data values

## Figures and Tables

**Table 1:**
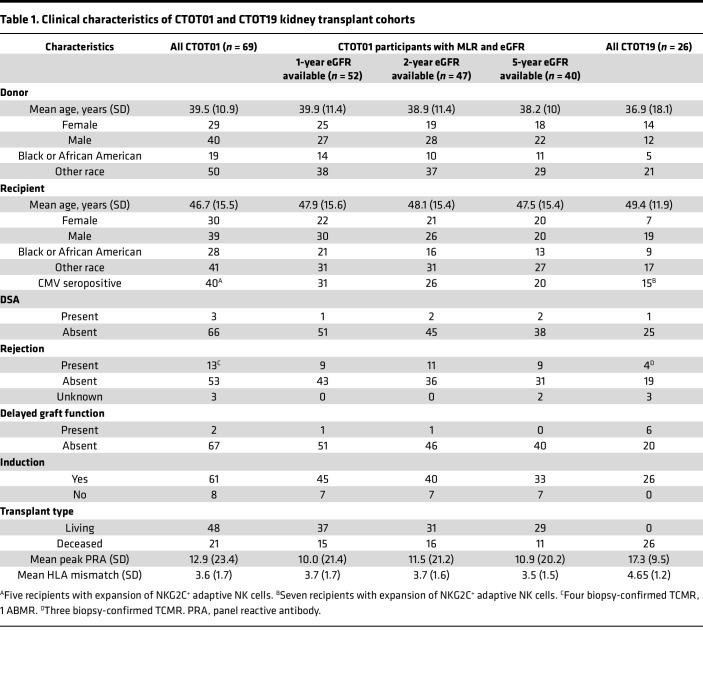
Clinical characteristics of CTOT01 and CTOT19 kidney transplant cohorts

## Version 1 10/10/2024

In-Press Preview

## Version 2 11/08/2024

Electronic publication
