# Supplementary material for: High-dimensional analysis of NK cells in kidney transplantation uncovers subsets associated with antibody-independent graft dysfunction
Source: JCI Insight. 2024 Nov 8;9(21):e185687. doi: 10.1172/jci.insight.185687 (PMC11601574; doi:10.1172/jci.insight.185687)
Supplement: Supplemental data [file jciinsight-9-185687-s056.pdf]

Supplemental Figure 1

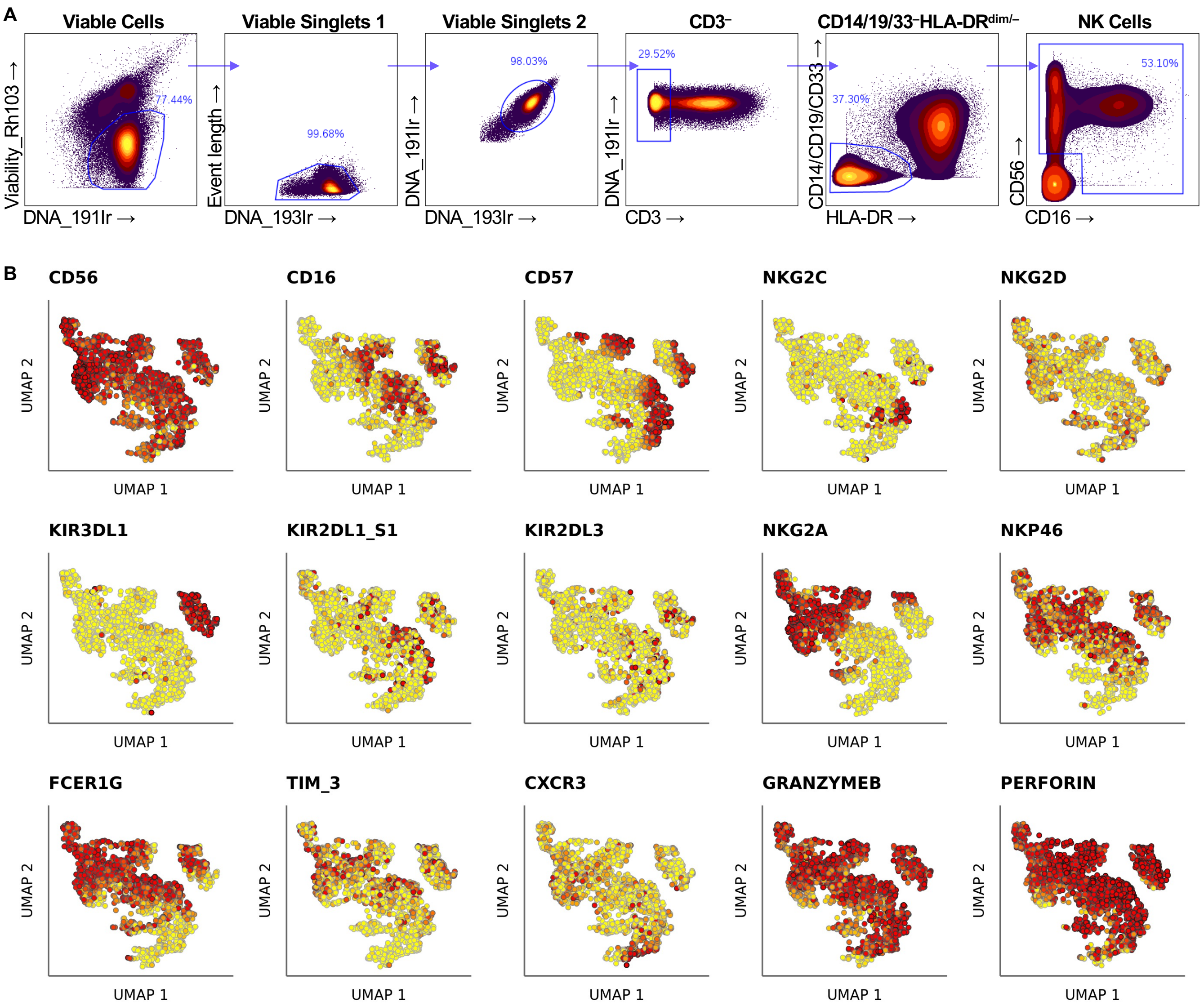

Supplemental Figure 1. Expression profile of NK cell markers is highly variable across subpopulations A Representative FACS plots show CyTOF gating strategy for total NK cells. **B** U-MAPs show mean signal intensity (MSI) and distribution of primary surface and intracellular markers that define key NK cell subsets.

Supplemental Figure 2

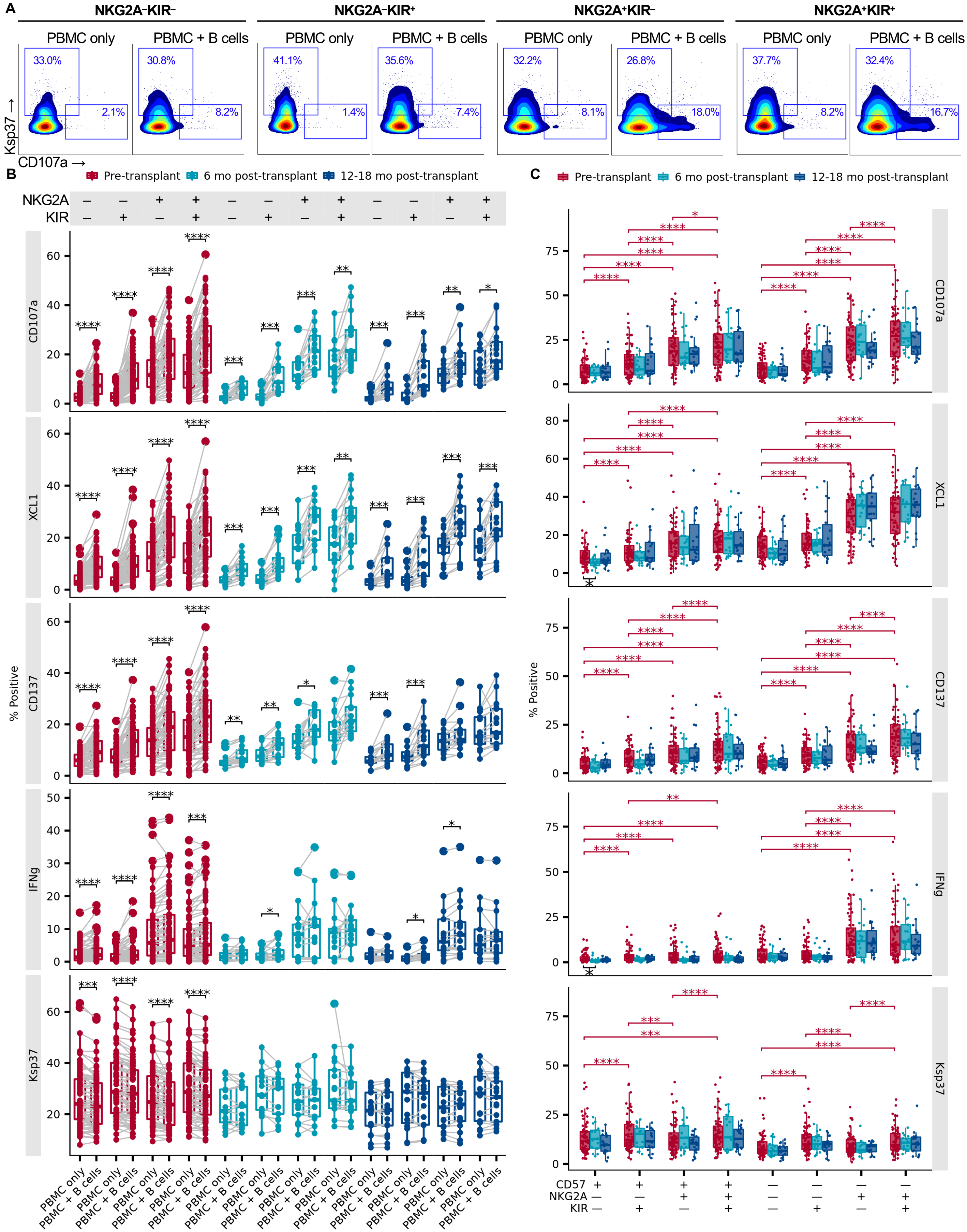

Supplemental Figure 2. NK cell alloreactivity is highly variable, transcends donor differences and is maintained post-transplant. A Representative FACS plots show gating of Ksp37⁺ and CD107a⁺ cells across NKG2A⁻KIR⁻, NKG2A⁻KIR⁺, NKG2A⁺KIR⁻, NKG2A⁺KIR⁺ NK cell subsets in unstimulated PBMC only condition and donor cell-stimulated condition. Allo-stimulation with donor cells induce loss of Ksp37 and increase in CD107a. **B** Subsets were defined by gating on CD56^{dim} NK cells followed by Boolean gating of educating inhibitory receptors, NKG2A, KIR3DL1, KIR3DL2, KIR2DL1 and KIR2DL3. Paired boxplots show change in percent positive of effector molecule at baseline and after 6-hour stimulation with kidney donor cells. **C** Subsets were further defined by CD57 expression. Boxplot shows percent positive of effector molecule in recipient NK cell subsets in response to 6-hour stimulation with kidney donor cells for which pre- (n=68) and post-transplant cells were available (n=34). Wilcoxon *p* values were adjusted for multiple testing with Bonferroni correction. ** $p < 0.01$, *** $p < 0.001$, **** $p < 0.0001$

Supplemental Figure 3

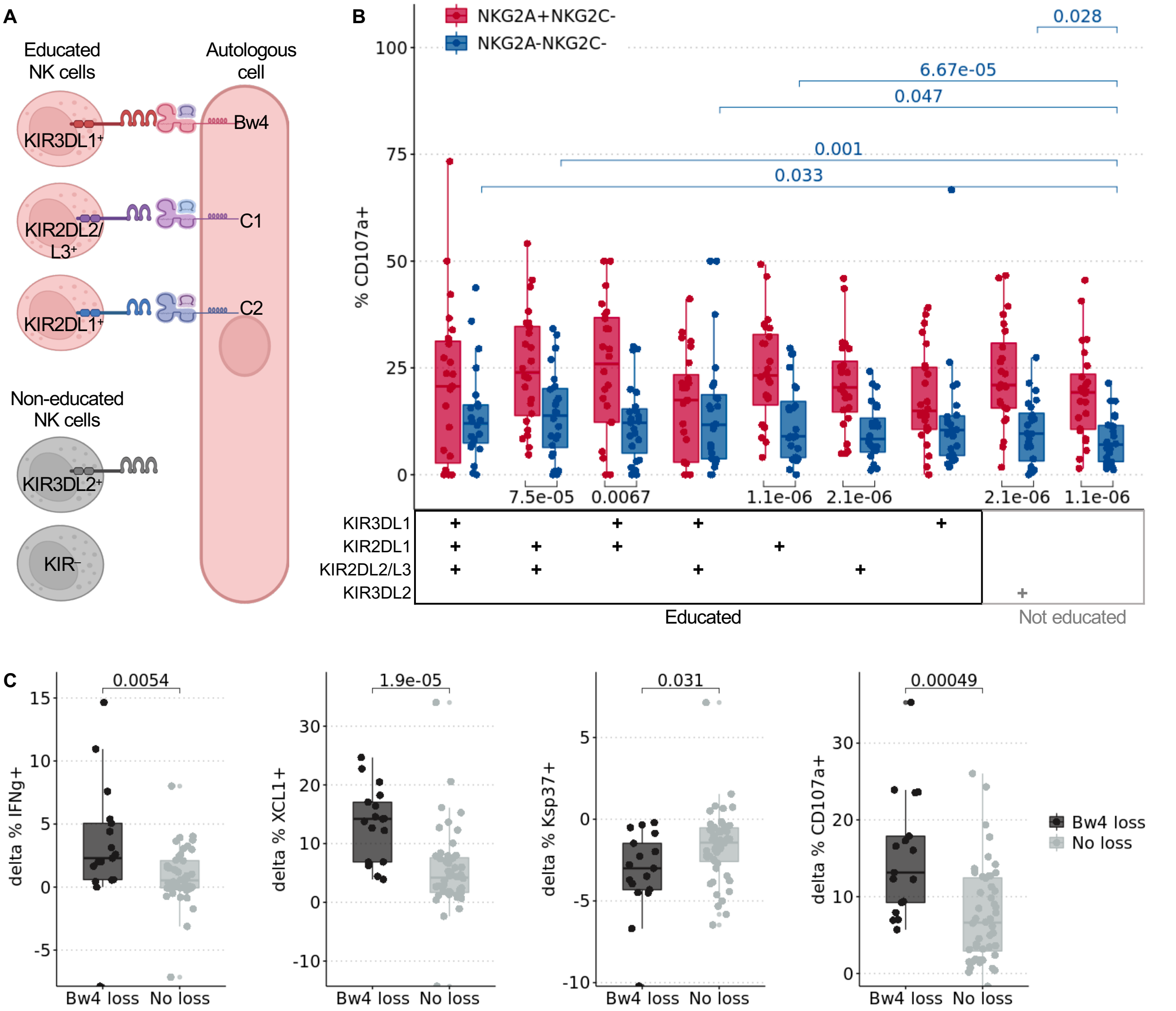

Supplemental Figure 3. NKG2A-educated and KIR-educated NK cells have stronger responses to allo-donor cells than non-educated NKG2A⁻ and KIR⁻ NK cells and respond to missing-self. A Schematic of KIR⁺ NK cells educated by Bw4, C1, and C1 presented on class I HLA. KIR3DL2⁺ are not educated due to lack of A3 and A11 KIR ligands. **B** NK cell subsets were defined by gating on CD56^{dim} NK cells followed by Boolean gating on educating inhibitory receptors, NKG2A, KIR3DL1, KIR3DL2, KIR2DL1 and KIR2DL3. Boxplot shows percent CD107a⁺ in pre-transplant NK cell subsets of recipients educated by Bw4, C1 and C2 KIR ligands (n=24) after stimulation with kidney donor cells. **C** Bw4 loss was defined as lower Bw4 copy number in transplant donor compared to recipient. No loss of Bw4 was defined as equal or greater copy number of Bw4 on HLA-A/B alleles in donor compared to recipient or recipient was *KIR3DL1*$^{-/-}$. Boxplots show change of percent effector molecules in KIR3DL1⁺ NK cells of recipients with loss (n=17) and no loss (n=48) of Bw4. *P* values reflect Wilcoxon test with Bonferroni correction.

Supplemental Figure 4

A

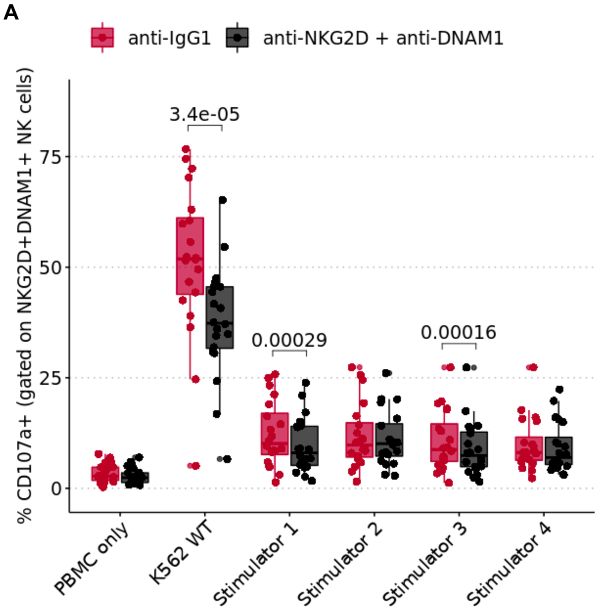

B

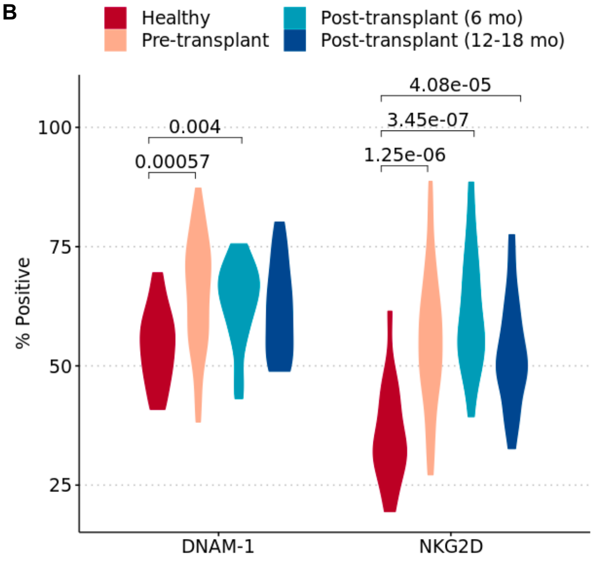

Supplemental Figure 4. NK cell alloreactivity was abrogated by blockade of activating receptors. A Activation of NKG2D⁺DNAM-1⁺ NK cells in healthy subjects with and without blockade of NKG2D and DNAM-1. **B** Expression of DNAM-1 and NKG2D in healthy and CTOT01 NK cells. *P* value reflects Wilcoxon test with Bonferroni correction.

Supplemental Figure 5

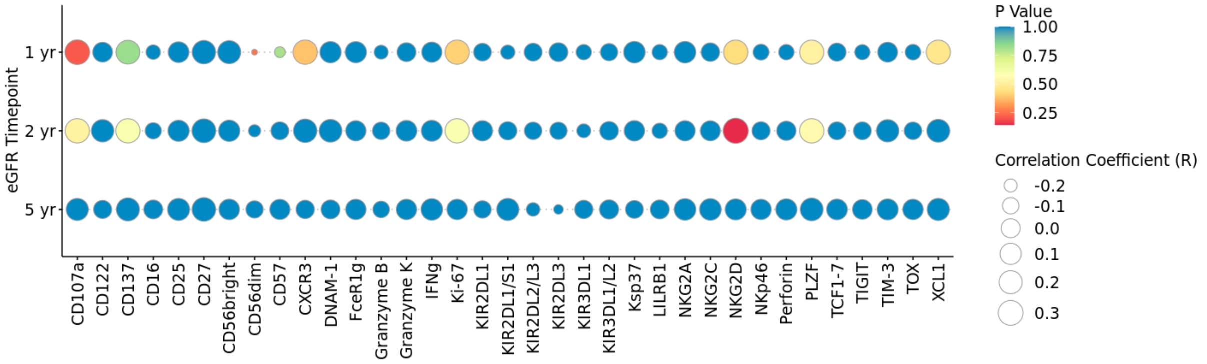

	1 year		2 year		5 Year	
	Adjusted P val	R	Adjusted P val	R	Adjusted P val	R
CD56dim	0.2546874	-0.2858489	1.0000000	-0.2165124	1	-0.0662254
CD56bright	1.0000000	0.2049105	1.0000000	0.1108497	1	0.0737740
KIR2DL1	1.0000000	-0.0509638	1.0000000	0.0589167	1	-0.0620869
KIR2DL3	1.0000000	-0.0069684	1.0000000	-0.0678343	1	-0.2609511
KIR3DL1	1.0000000	-0.1346598	1.0000000	-0.1760193	1	-0.0279603
KIR3DL1/L2	1.0000000	-0.0674032	1.0000000	-0.0147584	1	0.0081007
KIR2DL2/L3	1.0000000	-0.0457575	1.0000000	-0.0612891	1	-0.1859810
KIR2DL1/S1	1.0000000	-0.1679366	1.0000000	-0.0306480	1	0.1546353
IFNg	1.0000000	0.0550752	1.0000000	0.0934975	1	0.1240174
CD107a	0.2060103	0.2929950	0.5167011	0.2751565	1	0.1530107
Perforin	1.0000000	-0.1323662	1.0000000	0.0142795	1	0.1055885
Granzyme B	1.0000000	-0.1740611	1.0000000	-0.0613664	1	-0.0945328
Ksp37	1.0000000	0.1195967	1.0000000	0.0665379	1	-0.0450896
CD137	0.8279496	0.2424593	0.6026528	0.2690590	1	0.2042181
XCL1	0.4640281	0.2646128	1.0000000	0.1949798	1	0.1552410
CD25	1.0000000	0.0827615	1.0000000	0.1038435	1	0.1499942
LILRB1	1.0000000	-0.1356012	1.0000000	-0.1346216	1	-0.0017556
CD57	0.8072443	-0.2434668	1.0000000	-0.0450445	1	0.0556810
NKG2D	0.4291310	0.2674726	0.1431576	0.3215987	1	0.0996035
DNAM-1	1.0000000	0.1088204	1.0000000	0.1860821	1	-0.0032933
PLZF	0.5127462	0.2609178	0.5481981	0.2728271	1	0.1881770
TCF1-7	1.0000000	-0.0367976	1.0000000	-0.0443707	1	0.0816820
TIM-3	1.0000000	0.0408448	1.0000000	0.1656032	1	0.0960319
TIGIT	1.0000000	-0.1416303	1.0000000	-0.0471749	1	0.0322175
NKp46	1.0000000	-0.1169255	1.0000000	-0.0362309	1	0.0624468
NKG2A	1.0000000	0.1219388	1.0000000	0.0559600	1	0.1267347
Ki-67	0.4064993	0.2694374	0.5940981	0.2696311	1	0.0667811
CD27	1.0000000	0.2245498	1.0000000	0.2445340	1	0.2369151
CXCR3	0.3808516	0.2717828	1.0000000	0.2247517	1	-0.0254742
NKG2C	1.0000000	-0.0191608	1.0000000	0.0460293	1	0.1284774
FceR1g	1.0000000	0.1058017	1.0000000	0.0574559	1	0.0628619
Granzyme K	1.0000000	-0.0038365	1.0000000	0.0829012	1	0.0657245
CD122	1.0000000	0.0367720	1.0000000	0.1684389	1	-0.0336520
TOX	1.0000000	-0.1260139	1.0000000	-0.0570490	1	0.0643918
CD16	1.0000000	-0.1590597	1.0000000	-0.1042505	1	-0.0051008

Supplemental Figure 5. NK cell markers do not correlate with eGFR. Percent size of NK cell subset expressing surface and intracellular markers was determined by manual gating. Bubble plot and table show correlations between percent of marker in total NK cells and eGFR at 1-year (n=52), 2-years (n=47), and 5-years (n=40) in CTOT01. *P* values reflect Pearson correlation with Bonferroni correction for multiple comparisons.

Supplemental Figure 6

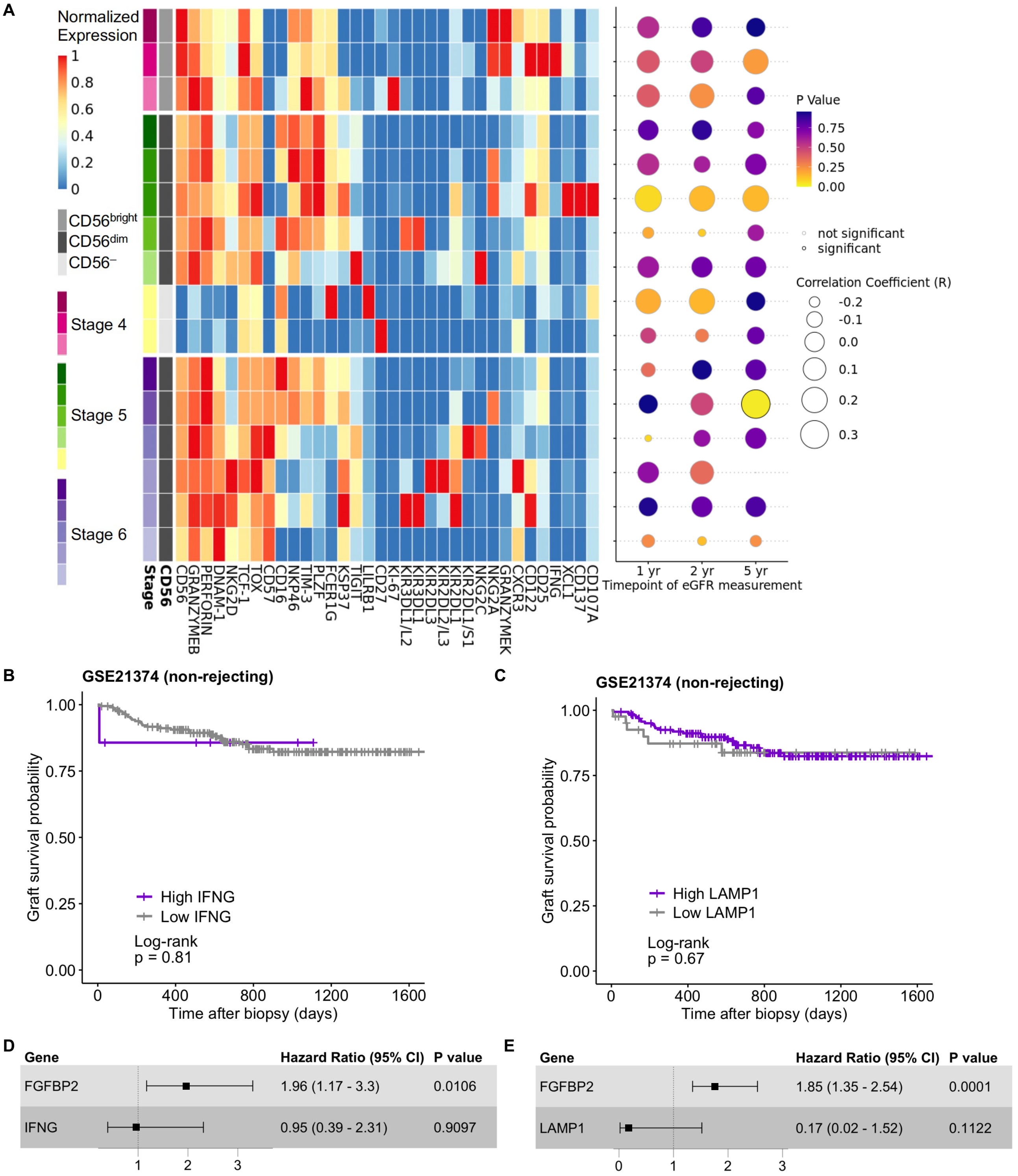

Supplemental Figure 6. NK subset abundance does not correlate with eGFR. A Heatmap shows median expression of CyTOF antibodies defining NK cell subsets defined by hierarchical clustering. Adjacent bubble plot shows correlations between NK cell subset size and eGFR at 1-year (n=52), 2-years (n=47), and 5-years (n=40) in CTOT01. Nominal *p* values reflect Pearson correlation. No correlations were significant after corrections for multiple comparisons. **B** Kaplan Meier plot shows time to graft failure in subset of non-rejecting recipients from GSE21374 with either high (n=7) or low (n=199) expression of *IFNG*. **C** Kaplan Meier plot shows time to graft failure in subset of non-rejecting recipients from GSE21374 with either high (n=164) or low (n=42) expression of *LAMP1*. High and low expression of *IFNG* and *LAMP1* were determined by StepMiner, as previously reported.(43) **D** Forest plot of cox regression model for the effect of *FGFBP2* and *IFNG* on time to graft failure in non-rejecting recipients from GSE21374 (n=206). **E** Forest plot of cox regression model for the effect of *FGFBP2* and *LAMP1* on time to graft failure in non-rejecting recipients from GSE21374 (n=206). *P* values in **B**-**C** were computed by Log-rank test.

Supplemental Table 1. Multivariate regression models of eGFR for CTOT01

Variable	Estimate	P Value
CTOT01		
eGFR at 1 year (regression model 1)		
Intercept	75.974	3.25E-07
**%Ksp37+**	**-3.831**	**0.0516**
Recipient Age	-0.362	0.0246
Donor Age	-0.352	0.1060
Donor Sex [Male]	11.779	0.0189
Living Transplant	22.581	0.0001
eGFR at 1 year (regression model 2)		
Intercept	73.716	4.91E-11
**%Ksp37+**	**-4.551**	**0.0614**
Thymoglobulin	-5.387	0.3851
HLA-A/B/DR mismatch	-1.070	0.5624
Rejection	-3.583	0.6616
Recipient Sex [Male]	2.783	0.6564
eGFR at 2 years (regression model 1)		
Intercept	58.601	2.12E-07
**%Ksp37+**	**-6.239**	**0.0028**
Recipient Age	-0.349	0.0511
Donor Sex [Male]	17.233	0.0036
Living Transplant	25.746	2.77E-05
eGFR at 2 years (regression model 2)		
Intercept	94.471	3.20E-09
**%Ksp37+**	**-5.994**	**0.0190**
Thymoglobulin	-0.831	0.9036
HLA-A/B/DR mismatch	-2.119	0.3404
Rejection	-0.757	0.9257
Donor Age	-0.441	0.1699
eGFR at 5 years (regression model 1)		
Intercept	73.130	4.14E-05
**%Ksp37+**	**-6.900**	**0.0409**
Thymoglobulin	-13.954	0.1210
HLA-A/B/DR mismatch	-2.063	0.4740
Rejection	1.856	0.8622
Living Transplant	1.709	0.8696
eGFR at 5 years (regression model 2)		
Intercept	74.469	0.0047
**%Ksp37+**	**-8.227**	**0.0254**
Recipient Age	0.078	0.8005
Donor Age	-0.466	0.3087
Recipient Sex [Male]	-4.934	0.5828
Donor Sex [Male]	8.096	0.3760

Supplemental Table 2. Multivariate regression models of eGFR for CTOT19

Variable	Estimate	P Value
CTOT19		
eGFR at 6 months (regression model 1)		
Intercept	55.812	9.17E-10
Delayed Graft Function	-1.314	0.8951
**%Ksp37+**	**-1.930**	**0.0261**
eGFR at 6 months (regression model 2)		
Intercept	64.750	3.01E-08
HLA-A/B/C mismatch	-2.969	0.1214
**%Ksp37+**	**-1.682**	**0.0423**
eGFR at 6 months (regression model 3)		
Intercept	57.435	4.40E-09
Rejection	-7.841	0.6460
**%Ksp37+**	**-2.338**	**0.0205**
eGFR at 6 months (regression model 4)		
Intercept	64.530	0.0020
Recipient Age	-0.194	0.6159
**%Ksp37+**	**-1.760**	**0.0549**
eGFR at 6 months (regression model 5)		
Intercept	62.191	1.46E-06
Donor Age	-0.215	0.4226
**%Ksp37+**	**-1.760**	**0.0990**
eGFR at 6 months (regression model 6)		
Intercept	53.059	1.47E-06
Recipient Sex [Male]	3.516	0.7116
**%Ksp37+**	**-1.959**	**0.0245**
eGFR at 6 months (regression model 7)		
Intercept	53.658	8.70E-07
Donor Sex [Male]	2.871	0.7719
**%Ksp37+**	**-1.785**	**0.0710**
eGFR at 2 years (regression model 1)		
Intercept	65.577	1.28E-09
Delayed Graft Function	-4.548	0.7032
**%Ksp37+**	**-2.073**	**0.0437**
eGFR at 2 years (regression model 2)		
Intercept	72.581	2.04E-07
HLA-A/B/C mismatch	-2.351	0.3147
**%Ksp37+**	**-1.869**	**0.0663**
eGFR at 2 years (regression model 3)		
Intercept	65.271	1.34E-08
Rejection	-8.866	0.6115
**%Ksp37+**	**-2.277**	**0.0574**
eGFR at 2 years (regression model 4)		
Intercept	75.704	0.0025
Recipient Age	-0.239	0.6063
**%Ksp37+**	**-1.858**	**0.0884**
eGFR at 2 years (regression model 5)		
Intercept	78.238	4.55E-07
Donor Age	-0.442	0.1632
**%Ksp37+**	**-1.325**	**0.2245**
eGFR at 2 years (regression model 6)		
Intercept	56.236	6.33E-06
Recipient Sex [Male]	11.900	0.2918
**%Ksp37+**	**-2.172**	**0.0325**
eGFR at 2 years (regression model 7)		
Intercept	67.329	4.31E-07
Donor Sex [Male]	-4.511	0.7042
**%Ksp37+**	**-2.285**	**0.0550**

Supplemental Table 3. CyTOF and flow cytometry antibodies

Metal	Antigen	Vendor	Catalog #
		**CyTOF Panel**	
89Y	CD45	Standard BioTools	3089003B
111Cd	LILRB1	BioLegend	333702
112Cd	CD8	BioLegend	301002
113Cd	CD38	Miltenyi	130-122-307
114Cd	CD3	BioLegend	300402
115In	CD14	BioLegend	301802 & 301843
115In	CD19	BioLegend	302202
115In	CD33	BioLegend	303402
116Cd	CD57	BioLegend	359602
141Pr	TCRvd2	BioLegend	331402
142Nd	CCL4	Miltenyi	130-095-212
143Nd	XCL1	R&D Sys	AF695
144Nd	Ksp37	BioLegend	custom
145Nd	NKG2D	Miltenyi	130-122-332
146Nd	DNAM-1	Miltenyi	130-092-479 & 130-126-485
147Sm	PLZF	R&D Sys	MAB2944
148Nd	KIR3DL1/L2	Miltenyi	130-126-489
149Sm	CD25	Standard BioTools	3149010B
150Nd	TCF-1/7	BioLegend	655202
151Eu	CCL5	R&D Sys	MAB278-100
152Sm	KIR3DL1	Miltenyi	130-092-555
153Eu	TIM-3	Standard BioTools	3153008B
154Sm	TIGIT	Standard BioTools	3154016B
155Gd	NKp46	Miltenyi	130-124-522
156Gd	KIR2DL2/L3	Miltenyi	130-122-346
158Gd	KIR2DL1	Miltenyi	130-122-279
159Tb	CD56	Miltenyi	130-108-016
160Gd	NKG2A	Miltenyi	130-122-329
161Dy	Ki-67	Standard BioTools	3161007B
162Dy	CD27	Standard BioTools	3162009B
163Dy	CXCR3	Standard BioTools	3163004B
164Dy	NKG2C	Miltenyi	130-122-278
165Ho	KIR2DL3	Miltenyi	130-122-280
166Er	KIR2DL1/S1	Miltenyi	130-122-345
167Er	FceR1g	EMD millipore	06-727
168Er	IFNg	Standard BioTools	3168005B
169Tm	Granzyme K	BioLegend	370502
170Er	CD122	Standard BioTools	3170004B
171Yb	Granzyme B	Standard BioTools	3171002B
172Yb	CD107a	Miltenyi	130-124-536
173Yb	CD137	Standard BioTools	3173015B
174Yb	TOX	Miltenyi	custom
175Lu	Perforin	Standard BioTools	3175004B
176Yb	CD4	Standard BioTools	3176010B
198Pt	HLA-DR	Miltenyi	130-122-299
209Bi	CD16	Standard BioTools	3209002B
	**Flow cytometry panel for profiling stimulator cell ligands**		
	HLA-A/B/C	BioLegend	311418
	MICA/B	BioLegend	320912
Panel 1	CD86	BioLegend	374214
	HLA-F	BioLegend	373208
	CD112	Miltenyi	130-122-770
	CD80	BioLegend	305225
	HLA-G	BioLegend	335912
Panel 2	ULBP1	R&D Systems	FAB1380A
	CD54	BioLegend	353117
	HLA-C	BD Biosciences	747594
	CD155	BioLegend	337634
	HLA-E	BioLegend	342604
Panel 3	PD-L1	BioLegend	329736
	ULBP2/5/6	R&D Systems	FAB1298G
	CD58	BioLegend	330918
Viability	Zombie NIR	BioLegend	423105
	**Flow cytometry panel for NK cell sorting and killing assay**		
	CD19	BioLegend	302244
	CD56	BioLegend	362550
	CD3	BioLegend	317346
	NKG2A	Miltenyi	130-128-163
	NKG2C	Miltenyi	130-117-398
	KIR3DL1/L2	Miltenyi	130-116-180
	KIR2D	Miltenyi	130-117-483
	CD107a	Miltenyi	130-111-624
	Ksp37	BioLegend	346603
	Zombie NIR	BioLegend	423106
	Propidium iodide	Life Technologies	P3566
